# A Comprehensive Review on *Graptopetalum paraguayense*’s Phytochemical Profiles, Pharmacological Activities, and Development as a Functional Food

**DOI:** 10.3390/plants14030349

**Published:** 2025-01-24

**Authors:** Varun Jaiswal, Hae-Jeung Lee

**Affiliations:** 1 Department of Food and Nutrition, College of BioNano Technology, Gachon University, 1342 Seongnam-daero, Sujeong-gu, Seongnam-si 13120, Republic of Korea; computationalvarun@gmail.com; 2 Institute for Aging and Clinical Nutrition Research, Gachon University, Seongnam-si 13120, Republic of Korea; 3 Department of Health Sciences and Technology, Gachon Advanced Institute for Health Sciences and Technology, Gachon University, Incheon 21999, Republic of Korea

**Keywords:** *Graptopetalum paraguayense*, phytochemicals, antioxidants, therapeutic, functional food, medicinal plant, horticulture plant

## Abstract

*Graptopetalum paraguayense* (*G. paraguayense*) is a succulent plant that has been used in traditional Chinese and Taiwanese medicine, mainly for antihypertensive and hepatoprotective activities. *G. paraguayense* is also used as an edible vegetable, which is considered a functional food. Different in vitro, in vivo, and clinical studies have highlighted the multiple pharmacological activities of *G. paraguayense*, which include anticancer, antibacterial, antiviral, antiasthma, antihypertensive, skin-whitening and anti-aging, anti-Alzheimer, neuroprotective, and hepatoprotective activities. Numerous studies revealed the antioxidant and anti-inflammatory potential of *G. paraguayense,* which may be the major contributing factor for multiple pharmacological activities and the protective effect of *G. paraguayense* on pancreatic, liver, lung, colon, and brain diseases. Initial safety studies on animal models also support the therapeutic candidature of *G. paraguayense*. The presence of numerous bioactive phytochemicals, especially polyphenols, and the identification of important disease targets of *G. paraguayense* emphasize its high therapeutic potential. The lack of a directional approach and limited in vivo studies limit the development of *G. paraguayense* against important diseases. Still, a compilation of pharmacological activities and target pathways of *G. paraguayense* is missing in the literature. The current review not only compiles pharmacological activities and phytochemicals but also highlights gaps and proposes future directions for developing *G. paraguayense* as a candidate against important diseases.

## 1. Introduction

*Graptopetalum paraguayense* E. Walther is a succulent plant that belongs to the family Crassulaceae. *Graptopetalum paraguayense* (*G. paraguayense*) is a perennial plant mainly used as an ornamental plant which is common in gardens, houses, and offices. *G. paraguayense* is native to Mexico, and the common names of *G. paraguayense* include ghost plant, opal grey, *Sedum weinbergii*, and mother-of-pearl-plant.

Succulent plants have a peculiar morphology that supports water storage, and several succulent plants are used in traditional medicine; e.g., *Lithops lesliei* is used to treat issues relating to female reproductive health, *Mesembryanthemum nodiflorum* is used to treat gastric disease, *Trianthema portulacastrum* is used to treat eye-related *issues*, *Opuntia humifusa* is active against diabetes, skin aging, and different types of cancer, and *Aloe vera* is used for multiple pharmacological activities [1,2,3].

*G. paraguayense* is an edible plant that is also used in traditional Chinese and Taiwanese herbal medicine for various purposes, including improving brain function, controlling blood pressure, serving as an anti-diabetic and a diuretic, promoting pain relief, treating infections, and treating liver-associated disorders [4,5]. The multiple uses of *G. paraguayense* in folk medicine have inspired researchers to study the plant to validate the various pharmacological properties of *G. paraguayense*. In vitro, in vivo, and clinical studies have emphasized the multiple pharmacological activities of *G. paraguayense* which include antibacterial, antiviral, antiasthma, antihypertensive, skin-whitening and anti-aging, anti-Alzheimer, neuroprotective, hepatoprotective, and anticancer activities against different types of cancer [6,7,8,9,10]. Its various pharmacological activities and use as an edible vegetable make *G. paraguayense* an excellent functional food.

**Figure 1:**
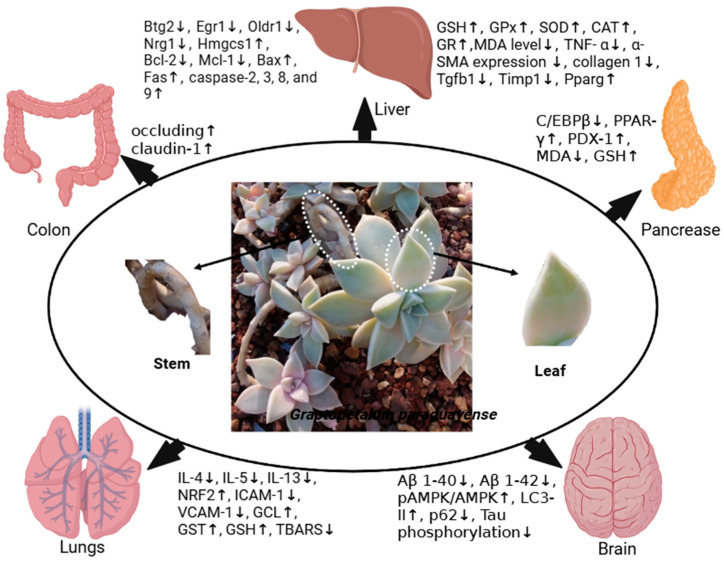
Protective effects of *Graptopetalum paraguayense* in different organs observed through in vivo studies (↑: upregulation; ↓: downregulation/inhibition).

These different pharmacological activities of *G. paraguayense* are in different priorities, e.g., the antioxidant and anti-inflammatory activities of *G. paraguayense* are believed to support other pharmacological activities of *G. paraguayense* including anti-diabetic, neuroprotective, and anticancer activities against different types of cancers [6,7,8,9,10]. Similarly, the antioxidant and anti-inflammatory activities of *G. paraguayense* also provide protective effects on different organs like the liver, colon, lungs, and brain (Figure 1). Several pharmacologically important phytochemicals, especially flavones, were also identified in *G. paraguayense,* which were reported to possess numerous medicinal properties. The presence of these phytochemicals again highlight the medicinal potential of *G. paraguayense* [11,12].

The different pharmacological activities of *G. paraguayense* are in various stages of development and have both general and specific challenges that need to be overcome for progress as a therapeutic and/or supplement against important diseases and/or conditions. Still, to the best of our knowledge, the literature does not have a review that compiles the pharmacological properties and phytochemicals of *G. paraguayense* to aid in the development of *G. paraguayense*’s pharmacological activities. The current review not only compiles the pharmacological activities and phytochemicals of *G. paraguayense* but also suggests future directions based on current gaps in the development of *G. paraguayense* as a therapeutic and/or supplement against important diseases.

## 2. Methods

A literature search was carried out through electronic literature databases such as Scopus, ResearchGate, and Web of Sciences. Various keywords and their combinations related to this study used for searching were *Graptopetalum paraguayense*, ghost plant, Sedum weinbergii, mother-of-pearl plant, pharmacological activities, anticancer, anti-parasite, anti-inflammatory, anti-diabetic, etc. Applicable documents related to the topic, such as review articles, research articles, theses, books, and book chapters in English from the search results of the databases were included in this study.

## 3. Botanical Description of *G. paraguayense*

*G. paraguayense* is a succulent plant that grows up to 1 foot in height and 2–3 feet in width. *G. paraguayense* has opalescent leaves with tones of grey, pink, and amethyst that are arranged in whorls and rosettes on stiff, creeping, or hanging stems (Figure 1). The leaves are fleshy, deltoid in shape, 1–3 inches long, and 0.5 to 1 inch wide, and they are thick flat triangular to spathulate leaves with pointed tips (Figure 1). The flowers of *G. paraguayense* are smaller in size (>1 inch) and have a star-shaped structure with five petals that are white with some red flecks. The flower inflorescence is a cyme, and the flowering time is spring. The plant is multi-stemmed with clumping habits and requires low maintenance (https://plants.ces.ncsu.edu/plants/graptopetalum-paraguayense/ (accessed on 10 December 2024)).

## 4. Toxicity Studies and Safety Profile of *G. paraguayense*

*G. paraguayense* has been used as an edible vegetable in Asian countries and as traditional medicine. However, the multiple pharmacological properties of *G. paraguayense* have created a need to study the safety of the *G. paraguayense* extract as a processed product.

Various in vitro and in vivo studies have shown the safety profile of *G. paraguayense*. In acute and sub-oral toxicity tests on male and female SD rats, the LD50 was found to be more than 5.0 g/kg as a single dose of 2.5 or 5.0 g/kg did not cause any significant change in body weight, relative organ weights, cumulative food or water intake, and hematological parameters [13]. Furthermore, no clinical sign or death was observed in animals with the treatment. Similarly, the 28-day treatment of SD rats with a 1.0 g/kg water extract of *G. paraguayense* did not cause any significant change in body weight, relative organ weights, cumulative food or water intake, and hematological parameters. Further, in a macroscopic study, all glands and organs were found to be morphologically normal. Similarly, organs such as the heart, liver, spleen, lungs, and kidneys were also found to be normal like in the control group in the histopathological analysis [13].

In male ICR mice, micronucleus tests revealed that *G. paraguayense* water extract of up to 5.0 g/kg in dose was found to be safe and did not alter the clinical profile or body weight of mice [13]. The micronucleated reticulocytes were in the normal range in all animals that consumed the water extract of *G. paraguayense*.

Chinese hamster ovary (CHO-K1) cells were used to assess the genotoxic potential of *G. paraguayense* extract. The chromosomal aberrations in CHO-K1 cells with the treatment of water extract of *G. paraguayense* revealed that the *G. paraguayense* extract was safe up to 1.2 mg/mL in terms of genotoxicity [13].

## 5. Phytochemicals of *G. paraguayense*

The plant’s phytochemicals are considered the main factor responsible for their pharmacological activities. The phytochemical profile of plants not only varies in different parts of the plants (like the roots, leaves, and stems) but also depends on the surrounding environment and phase of the plant (such as the premature, intermediately mature, and mature phases). Medicinal activities from traditional usage have been the main reason behind analyses of the phytochemical profile of *G. paraguayense*. The phytochemical analysis of *G. paraguayense* has mostly been conducted through pharmacological studies to explore the phytochemicals responsible for their pharmacological activity.

To correlate phytochemical content with antioxidant activity, antioxidant activities of leaf extracts in different solvents, i.e., water (GPLWE), 50% ethanol (GPL50E), and 95% ethanol (GPL95E) were studied along with analysis of total phenolic content (TPC) and total anthocyanin content (TAC). The TPCs of GPLWE, GPL50E, and GPL95E were 22.7 ± 1.10, 34.0 ± 1.3, and 11.0 ± 0.4 mg (gallic acid equivalent/g), respectively. Similarly, the TACs of GPLWE, GPL50E, and GPL95E were 0.54, 1.29, and 0.03 μmol/g, respectively [5]. Importantly, the studied pharmacological activity—i.e., antioxidant activity—was in correlation with TAC, and TPC which was highest for GP50E. Later, TAC and TPC were also analyzed for stem extract in different solvents, i.e., water (GPSWE), 50% ethanol (GPS50E), and 95% ethanol (GPS95E). The study revealed the TPCs of GPSWE, GPS50E, and GPS95E were 56.4 ± 2.2, 45.9 ± 0.2, and 23.8 ± 2.0 mg (gallic acid equivalent/g), respectively. Similarly, the TAC was 0.30 ± 0.02, 0.15 ± 0.00, and 0.12 ± 0.02 μmol/g, respectively.

Similarly, the identification of individual phytochemicals was also conducted to identify the components responsible for pharmacological activity. An analysis of phytochemicals present in the leaf extract of *G. paraguayense* was conducted after observing the protective effect of leaf extract on brain injury [14]. The role of polyphenols in protecting the brain inspired researchers to study the presence of polyphenolic compounds through an HPLC-based method. Compound identification was based on a combination of retention time and spectral matching via comparison with known standards. The active ethanol extract of the leaf and its ethyl acetate extract revealed the presence of four pharmacologically active compounds: gallic acid, genistin, daidzin, and quercetin [14]. The amount of gallic acid was found to be the highest among the four phytochemicals found in the extracts (Table 1).

The best harvesting time according to the content of phytochemicals in different maturity phases of the plant was studied. Across the different maturity phases (immature, intermediately mature, and mature), the highest flavonoid and phenolic content was observed in immature plant leaves. The fermentation of leaves could increase the phenolic and flavonoid content in all the plant leaves studied, i.e., immature, intermediately mature, and mature leaves [12].

The HPLC-based method was used to analyze the difference in the components of organic acids such as oxalic acid and hydroxybutanedioic acid and phenolic compounds (such as caffeic acid, chlorogenic acid, rutin, quercetin, and gallic acid) in immature, intermediately mature, and mature plants. The immature *G. paraguayense* plants had the highest content of phytochemicals, which decreased with maturity level, i.e., the content of chlorogenic acid, rutin, quercetin, and gallic acid was found to be decreased with maturity. After achieving antioxidant and hepatoprotective activities in 50% ethanolic extract of leaves, the HPLC-based identification of the extract revealed that the major organic phytochemicals present in the extract were flavonoids. Further, the HPLC phytochemical profile of an extract studied via relative retention times using authentic standards detected gallic acid, flavone, genistin, daidzin, and quercetin [15].

**Table 1:** Phytochemicals reported in *Graptopetalum paraguayense*.

Sr. No.	Compound Name	Amount (µg/g)	Type of Extract/Fraction	Part of Plant	Pharmacological Activity	References
1	Genistin	0.27 ± 0.08 ^#^	Ethyl acetate fraction of extract	Leaf	PNS	[14]
2	Daidzin	1.16 ± 0.09 ^#^	Ethyl acetate fraction of extract	Leaf	PNS	[14]
3	Quercetin	7.8 ± 0.1	Aqueous extract	Immature plant leaf	PNS	[12]
		4.3 ± 0.1	Aqueous extract	Intermediately mature plant leaf		
		2.8 ± 0.1	Aqueous extract	Mature plant leaf		
4	Gallic acid	11.0 ± 1.5	Aqueous extract	Immature plant leaf	Antioxidant activity	[12]
		8.4 ± 1.1	Aqueous extract	Intermediately mature plant leaf		
		6.8 ± 0.8	Aqueous extract	Mature plant leaf		
5	Oxalic acid	27.2 ± 1.8	Aqueous extract	Immature plant leaf	PNS	[12]
		22.3 ± 2.1	Aqueous extract	Intermediately mature plant leaf		
		20.4 ± 1.8	Aqueous extract	Mature plant leaf		
6	Hydroxybutanedioic acid	17.6 ± 0.6	Aqueous extract	Immature plant leaf	PNS	[12]
		15.5 ± 0.7	Aqueous extract	Intermediately mature plant leaf		
		14.3 ± 0.5	Aqueous extract	Mature plant leaf		
7	Rutin	0.8 ± 0.1	Aqueous extract	Immature plant leaf	PNS	[12]
		0.5 ± 0.1	Aqueous extract	Intermediately mature plant leaf		
		0.4 ± 0.1	Aqueous extract	Mature plant leaf		
8	Quercetin 3-O-[6″-(3-hydroxyl-3- methylglutaroyl)]-β-d-glucopyranoside	4.8	Methanolic extract	Leaf	Antioxidant activity	[11]
9	Kampferol 3-O-[6″-(3-hydroxyl-3-methylglutaroyl)]-β-d-glucopyranoside	5.7	Methanolic extract	Leaf	Antioxidant activity	[11]
10	Quercetin 3-O-[6″-(3-hydroxyl-3-methylglutaroyl)- 2″-acetyl]-β-d-glucopyranoside	4.3	Methanolic extract	Leaf	Antioxidant activity	[11]
11	Kampferol 3- O-[6″-(3-hydroxyl-3-methylglutaroyl)-2″-acetyl]-β-dglucopyranoside	2.5	Methanolic extract	Leaf	Antioxidant activity	[11]
12	Isoquercetin	NP	Methanolic extract	Leaf	Antioxidant activity	[11]
13	Kaempferol 3-O-β-d-glucopyranoside	NP	Methanolic extract	Leaf	Antioxidant activity	[11]
14	Kaempferol	NP	Methanolic extract	Leaf	Antioxidant activity	[11]
15	Isoquercitrin-6-(3-hydroxy-3-methylglutarate)	90.52 ± 2.69 ^#^	Aqueous extract	Leaf	Antioxidant activity	[16]
16	Astragalin-6-(3-hydroxy-3-methylglutarate)	23.23 ± 0.5 ^#^	Aqueous extract	Leaf	Antioxidant activity	[16]
17	Isoquercitrin -2-acetyl-6-(3-hydroxy-3-methylglutarate)	77.35 ± 0.55 ^#^	Aqueous extract	Leaf	Antioxidant activity	[16]
18	Astragalin-2-acetyl-6(3-hydroxy-3-methylglutarate)	40.62 ± 0.29 ^#^	Aqueous extract	Leaf	Antioxidant activity	[16]

^#^ mg/g; NP: not provided; PNS: pharmacological activity was not studied in the study.

## 6. Pharmacological Activities of *G. paraguayense*

The use of *G. paraguayense* in traditional medicines was the initial motivation for analyzing its pharmacological activities. Subsequently, the anti-inflammatory activities have also proven to be hepatoprotective, pushing studies to analyze the positive effect of *G. paraguayense* on diseases associated with other organs such as the lungs, brain, colon, and pancreas. Various in vitro, in vivo, and clinical studies have revealed the different pharmacological activities of *G. paraguayense*.

### 6.1. Anticancer Effects

Herbs account for the widest source of potential medicine worldwide for almost all types of diseases including cancer [17,18]. The anticancer activity of *G. paraguayense* has been studied in both in vitro and in vivo experiments. Cell line studies have shown the anticancer activities of *G. paraguayense* against different types of cancer including skin, colon, and liver cancer [19].

#### 6.1.1. In Vitro Anticancer Effects

The anticancer activity of water extract from the stem of *G. paraguayense* was selected over ethanolic extracts due to its high antioxidant activity, TAC, and TPC. The anti-proliferative activity of water extract was studied in the human hepatocellular carcinoma cell line, i.e., HepG2. The dose-dependent inhibition of HepG2 cells by water extract was observed in a staining-based assay (Table 2). Cell cycle and apoptosis analysis also revealed that the extract treatment increased the population of G0/G1 and apoptotic cells via flow cytometry analysis. The cell cycle and apoptosis analysis also revealed that the extract treatment increased the population of G0/G1 and apoptotic cells via flow cytometry analysis [19]. The study suggested further molecular studies may be conducted to explore the molecular mechanism crucial to the anti-proliferative activity of the extract.

In another study, the anticancer activity of *G. paraguayense* was studied against liver cancer through liver cancer cell lines and animal models of liver cancer. Various extracts from the leaves of *G. paraguayense* were prepared using water, acetone, methanol, ethanol, 70% ethanol, 50% ethanol, 100% DMSO, and 30% DMSO [20]. The anti-proliferative activities of these different extracts were studied on two liver cancer cell lines, i.e., Huh7 and Mahlavu cells. The most active extract was found to be the 30%DMSO extract (30DE). The expression of oncogenes such as AURKA, AURKB, and FLJ10540 is known to be over-expressed in the hepatocellular carcinoma cells (HCCCs) and was found to be suppressed in a Western blot (WB) analysis of protein expression in HCCC cell lines such as Huh7 and HepG2 with the treatment of 30DE. Further, the study revealed that the protein expression levels of these oncogenes were decreased during the interphase and metaphase, which had been high during mitosis. The 30DE was further fractioned into four fractions to identify the most active fraction. The WB analysis revealed that the third fraction of 30DE (30DE3) was able to effectively suppress the expression of AURKA and AURKB in HepG2 cells. Additionally, the expression of AURKA and FLJ10540 was found to be suppressed by 30DE3 in all the other three HCCCs used in the study, i.e., Huh7, Mahlavu, and PLC5 cells. An NMR spectral analysis revealed that the presence of total tannin content was approximately 68% in 30DE3. High anti-proliferative activities of 30DE3 were also observed against the three studied HCCCs, i.e., Huh7, Mahlavu, and PLC5 cells, in a dose-dependent manner. The effect of 30DE3 on the intrinsic apoptotic pathway through mitochondrial membrane permeabilization (MMP) was studied. MMPs were decreased in both Mahlavu and Huh7 cells in the treatment group. In a concentration-dependent manner, the generation of reactive oxygen species (ROS) and intracellular peroxide was increased in the 30DE3-treated HCCCs. The free radical scavenger catalase was able to reduce 30DE3-induced cytotoxicity in HCCCs.

Further, in the immune blotting study, the levels of cleaved caspase-9, caspase-3, and PARP proteins were enhanced in the treatment, and suppression of the expression of anti-apoptotic proteins such as BCL2 and BCL-XL was observed. The expression of intrinsic pathway proteins suggested the involvement of the intrinsic apoptotic pathway in the 30DE3 treatment through ROS generation, causing caspase-dependent apoptosis. Similarly, the cell-proliferation-associated AKT pathway was found to be inhibited with 30DE and 30DE3 in the treatment group in HCCCs, i.e., Huh7 and Mahlavu cells. In the 30DE3 treatment, the level of phosphorylated AKT was suppressed and increased the levels of phosphatase and tensin homolog (PTEN), which is the negative regulator of PI3K-/AKT-dependent signaling. Additionally, to study the synergistic effect of *G. paraguayense* extracts, the anti-proliferative activities of extracts with sorafenib (an FDA-approved drug for the treatment of hepatocarcinoma) were studied. The results of the combination of *G. paraguayense* extracts such as 30DE3 and sorafenib synergistically inhibit the proliferation of Huh7 cells.

After compositional analysis of extract of leaves in water (LEW) and its 72% ethanol precipitate-containing macromolecules (LEWP), a preliminary study evaluating anti-proliferative activity against colon cancer cells was conducted. In the study, anti-proliferative activity on colon cancer cells was analyzed through the 3-(4,5-dimethylthiazol-2-yl)-2,5-diphenyl-tetrazolium bromide (MTT) assay. The study on the inhibition of Caco-2 cells (human colon adenocarcinoma cells) by the LEW and LEWP revealed the dose-dependent inhibition of Caco-2 cells, which was higher for LEWP [6].

To study the effect of *G. paraguayense* on skin cancer, the anticancer activity of 50% methanol extract prepared from the leaves of *G. paraguayense* was studied against melanoma cells (A375.S2 cells). Dose- and time-dependent growth inhibition of melanoma cells by the extract was observed in a flow-cytometry-based experiment. Anatomical changes were also observed in melanoma cells at a ≥125 μg/mL concentration [21]. The cell cycle distribution revealed cell cycle arrest (at the G2/M phase) and an increase in apoptotic cells in a dose-dependent manner. WB analysis also showed the expression of proteins related to the G2/M phase such as CDC2, CDC25c, Cyclin A, and Cyclin B were decreased, and the expression of proteins such as CHK1, CHK2, Weel, p21, and p53 were increased in the treatment group. DNA damage through chromatin condensation and an increase in caspase-3 activity were also observed in the treatment groups in further experiments. A study on calcium signaling revealed an increase in the intracellular levels of Ca^2+^, GRP78, GADD153, and caspase-7. Mitochondria-dependent apoptosis signaling was also found to be affected in the treatment. The loss of MMP (ΔΨm) in A375.S2 cells was observed with an early time of (0.5–2 h) exposure to the extract. The protein expression of apoptosis-inducing factor (AIF), Bax, and caspase-9 was enhanced in the treatment group, but the expression of Bcl2 was suppressed. The translocation of AIF, GADD153, and Endo G toward the nucleus was enhanced in the treatment, and the release of cytochrome c from mitochondria to cytosol was also observed in the treatment. The antioxidant effect of the treatment was also studied. The reactive oxygen species (ROS) level in the treated cells was found to be significantly suppressed as compared to the control cells, revealed through the H2DCF-DA (ROS indicator). The protein expression of antioxidant enzymes such as catalase and superoxide dismutase (SOD) was significantly decreased in the treated cells. Further, the activities of the cellular antioxidant enzymes including catalase, SOD, and GPx activities were also significantly suppressed in the treatment. Similarly, the GSH level was found to be decreased and the MDA level was found to be increased in the treated cells. The study proposed that the treatment can increase the Bax/Bcl-2 ratio and Ca^2+^ levels, change the MMP to trigger the release of cytochrome c, and subsequently induce the processing of procaspase-9 and procaspase-3, leading to DNA fragmentation; these are among the important mechanisms for the anticancer effects of *G. paraguayense*.

#### 6.1.2. In Vivo Anticancer Effects

After an in vitro cell line study of different liver cancer cell lines, an animal study on carcinogenic chemical diethylnitrosamine (DEN)-induced chronic liver disease was conducted [20]. Male Wistar albino rats were used to study the anticancer effects of *G. paraguayense* extracts (in low and high doses) and their 30DE3 fraction. The degree of cirrhosis and anticancer effects in these animals was evaluated by measuring bile flow rates, the spleen-to-body-weight ratio (SW/BW), the expression of α-SMA, and the liver tumor (Table 3). The bile flow rate was significantly increased in the high-dose *G. paraguayense* extract treatment group, which reflects an improvement in liver function. Similarly, the SW/BW ratio was significantly reduced in the high-dose *G. paraguayense* extract treatment group, which had been significantly higher in the animals due to DEN administration. The expression of α-SMA was significantly reduced in the high-dose *G. paraguayense* extract treatment group, which had also been higher in the DEN administration group. The collagen content of the liver was increased due to DEN administration, which indicated cirrhosis of the liver. The collagen content of the liver was found to be significantly decreased in the animals of all treatment groups, i.e., high and low doses of *G. paraguayense* and 30DE3 fraction groups. In the histopathology analysis of the liver, the numbers of tumors (per mm^3^) were found to be significantly decreased in groups administered high and low doses of *G. paraguayense* and 30DE3 fraction. The unevenness on the surfaces of the livers was also found to be significantly decreased in all treatment groups [20].

### 6.2. Anti-Alzheimer Disease Effects of G. paraguayense

Alzheimer’s disease (AD) is the most common neurodegenerative disorder with no effective treatment. Herbal medicines against AD and related diseases have been studied, showing positive outcomes [22]. The anti-AD effects of *G. paraguayense* were also studied through in vitro and in vivo studies. The inhibition of Aβ (both Aβ40 and Aβ42) and phosphorylation of Tau were studied in vitro studies, which are considered main features in AD pathology [23].

#### 6.2.1. In Vitro Anti-AD Effects of *G. paraguayense*

In view of the different pharmacological properties of *G. paraguayense,* and in silico gene set enrichment analysis (GSEA), the anti-AD activities of *G. paraguayense* were studied on cells derived from the AD patients [24]. Human-induced pluripotent stem cells from AD patients bearing the ApoE ε4 polymorphism and the FAD mutation were used in the study. These cells were differentiated into their corresponding neurons (ADNs) to study the anti-AD activity of the 30DE3 fraction of *G. paraguayense*. The ADNs showed a high level of pathological markers of AD, including the phosphorylation of Tau protein and extracellular accumulation of Aβ. 30DE3 fraction of *G. paraguayense* was subjected to a toxicity study through lactate dehydrogenase (LDH). There was no significant increase in LDH response even at the highest dose of the fraction, i.e., 50 μg/mL. The treatment of the 30DE3 fraction resulted in the decreased secretion of Aβ40 and Aβ42 from the ADN cells. Similarly, the treatment of the 30DE3 fraction resulted in the suppression of Tau phosphorylation at Ser214 and Ser396 locations, which was higher in the case of Ser396 [24]. Further studies on an expanded population of AD-derived cell lines from AD patients resulted in similar suppression of Tau phosphorylation. The study suggested the *G. paraguayense* as a potential drug candidate for AD.

In an anti-aging and age-related disease study, the anti-AD effects of the 30DE3 fraction of *G. paraguayense* were studied through in vitro and in vivo experiments. Before studying the effect of the 30DE3 fraction on SH-SY5Y-APP_695_ cells for Aβ40 and Aβ42 secretion, the cytotoxicity study revealed that the 30DE3 fraction had no toxicity on SH-SY5Y-APP_695_ cells at the highest dose used in the study. The treatment of the 30DE3 fraction resulted in the decreased secretion of Aβ40 and Aβ42 from the SH-SY5Y-APP_695_ cells at 50 μg/mL in an enzyme-linked immunosorbent assay (ELISA) experiment. Further, to explore the molecular pathways behind anti-AD activities, a gene set enrichment analysis (GSEA) was conducted on the genes identified to be differentially expressed through L1000 expression profiling of HT29 cells treated with 5 μg/mL 30DE3 fraction. In the GSEA analysis, a significant number of genes were enriched in AD, Huntington’s disease (HD), and AMPK signaling pathways, which suggested the role of AMPK activation in the anti-AD activities of 30DE3 fraction [9].

To further confirm the role of the AMPK activation pathway, glial U87 cells were used to study the effect of 30DE3 fraction on the phosphorylation of AMPK [9]. The result from WB experiments showed that the activation of AMPK increases in the pAMPK/AMPK ratio [9].

Autophagy dysregulation is also considered an important aspect of AD pathology. Thus, autophagy was also studied on glial (U87) and neuronal (SH-SY5Y-APP_695_) cells [9]. The turnover number of the marker of the autophagosomal membrane, i.e., microtubule-associated protein 1A/1B-light chain 3 (LC3), was studied on both cells. The increase in LC3-II was observed in 30DE3-fraction-treated cells via WB analysis, which indicated an increase in the autophagy. Additionally, a reduction in the protein level of p62 was observed in 30DE3-fraction-treated SH-SY5Y-APP_695_ cells, which supports the autophagy flux in the treatment [9].

#### 6.2.2. In Vivo Anti-Alzheimer Disease Effects of *G. paraguayense*

After achieving significant suppression of secretion of Aβ40 and Aβ42 from the SH-SY5Y-APP_695_ cells by 30DE3 fraction, an animal study on APPswe/PS1dE9 (APP/PS1) double-transgenic mice was conducted to study the anti-AD effects of the fraction [9]. After 30 days of 30DE3 fraction treatment, a significant reduction (48%) in the deposition of Aβ in the cerebral hemisphere of APPswe/PS1dE9 mice was observed in the treatment group as compared to the control group through a thioflavin-S (ThS) fluorescent staining experiment [9]. Further, the ELISA analysis revealed a significant reduction in both soluble and insoluble Aβ1-40 levels in the cerebral cortex of the treatment group animals. Further, to explore the molecular pathways behind the anti-AD activities of the fraction, the AMPK signaling pathway was studied through WB analysis. The AMPK signaling pathway is known to be dysregulated in the brains of human AD patients and AD mouse models. In the experiment on APPswe/PS1dE9 mice, the suppression of the AMPK signaling pathway via a decrease in both pAMPK and AMPK was observed in the cerebral cortex of the brain. The fraction treatment restores the levels of both pAMPK and AMPK in the cerebral cortex of APPswe/PS1dE9 mice [9].

Further, the autophagy activity of the 30DE3 fraction was studied on the *Caenorhabditis elegans*, which may suggest that the autophagy activity of the fraction is evolutionarily conserved [9]. The transgenic *Caenorhabditis elegans* carrying GFP::LGG-1, which represents the activation of autophagy, was treated with the fraction. The level of GFP::LGG-1 puncta was significantly increased after two days of treatment, which indicated the activation of autophagy in the worm. The role of the master transcription factor of autophagy, HLH-30/TFEB, was also studied on transgenic *Caenorhabditis elegans*. The treatment of the 30DE3 fraction enhanced the nuclear translocation of HLH-30/TFEB; this was observed through the green fluorescent protein (GFP) fluorescence-based method [9].

### 6.3. Anti-Aging Effects of G. paraguayense

Inspired by the positive effects of *G. paraguayense* on age-related diseases, researchers have continued studying the anti-aging effect of 30DE3 fraction on *Caenorhabditis elegans* [9]. The transgenic *Caenorhabditis elegans* carrying 35 polyglutamine repeats (Q35) was used in the study to analyze the effect of the 30DE3 fraction on the mobility of *Caenorhabditis elegans* through a thrashing assay. The transgenic *Caenorhabditis elegans* was treated with 30DE3 fraction from the L4 larval stage. The adults were able to maintain 70% mobility on day 5 in the fraction-treated groups, and the vehicle control group was able to maintain only 30% mobility compared to day 1. Importantly, the effect of the 30DE3 fraction was studied on the lifespan of wild-type N2 *Caenorhabditis elegans*. The study revealed that the treatment significantly increased the lifespan of the animals, by up to 16%. To further explore the role of the FOXO transcription factor, DAF-16, in the improvement in lifespan, DAF-16 mutant *Caenorhabditis elegans* was subjected to the treatment. DAF-16 is involved in different longevity-related pathways such as germline and insulin/IGF-1 signaling pathways. The 30DE3 fraction treatment was able to increase the lifespan of DAF-16 mutant *Caenorhabditis elegans,* similarly to the wild type, suggesting that the enhancement in lifespan by the fraction is independent of the DAF-16 [9].

### 6.4. Antihypertensive Activities of G. paraguayense

The hypertensive activity of *G. paraguayense* has been reported from traditional usage and validated through in vitro, in vivo, and clinical studies [7].

#### 6.4.1. In Vitro Antihypertensive Activities of *G. paraguayense*

Researchers evaluated the antihypertensive activity of *G. paraguayense* extracts through the inhibition of the angiotensin-converting enzyme (ACE), which a plays crucial role in blood pressure regulation [25,26]. The different extracts of leaves from the *G. paraguayense* were prepared in water and 50 and 95% ethanol to achieve optimal activity. The FAPGG substrate-based assay for ACE inhibition and a positive control (captopril, a known ACE inhibitor) were used to study the antihypertensive activity of *G. paraguayense* extracts. The effective dose-dependent inhibition of ACE activity was observed for extracts of *G. paraguayense,* which was higher in the ethanolic extracts. The 95% ethanol extract had the highest ACE inhibition activity in the study [7]. An enzyme kinetics analysis for ACE inhibition conducted through Lineweaver–Burk plots revealed the mixed-type inhibition of ACE by the extracts. ACE is a metalloenzyme having Zn in its catalytic site, which is crucial for its activity. Further, the introduction of ZnCl_2_ in the inhibition study reduced the inhibition of the extract significantly. It suggested that the ACE inhibition by the extract may be due to the chelation of ZN^2+^ ions.

#### 6.4.2. In Vivo Antihypertensive Effects of *G. paraguayense*

After achieving in vitro antihypertensive activity in *G. paraguayense* extract, the researcher explored the antihypertensive activity of 50% ethanol extract in an animal study. Spontaneously hypertensive rats (SHRs) and age-matched normal Wistar Kyoto (WKY) rats were used in the study. Control and treatment groups with both types of animals were used to provide saline and saline +2.5 g/kg extract, respectively. In the SHR control group, a significant increase in the SBP, DBP, and MBP was observed with time, but in the treatment group, the DBP and MBP did not increase during the experiment period, even though a significant decrease in SBP was observed. Further, ACE activity in plasma, the kidney, and the lungs was found to be increased in the SHR control group to a higher level than the WKY rat control group, while there was no significant increase in the ACE activity in the SHR treatment group [27].

Considering the importance of antioxidant activity in hypertension, the antioxidant status was also analyzed in studied animals. The total antioxidant status (TAS) of plasma was found to be significantly lower in the SHR control group than the SHR treatment group. The highest MDA levels were observed in the brain, liver, and heart of the SHR control group, and these levels were not significantly changed in all other groups. The GSH levels in the heart and brain tissues of the SHR treatment group were significantly higher than those in the SHR control group. Similarly, in heart, brain, and liver tissues, the α-tocopherol content in the SHR treatment group was significantly higher than that in the SHR control group. In antioxidant enzyme activities, the catalase and GPx activities were lowest in all the liver, brain, and heart tissues [27].

The SHR control group had the lowest GPx and catalase activities in all the tested tissues (*p* < 0.05), while GPx and catalase activities in the SHR-GE50 group did not differ from those in the WKY rat groups (*p* > 0.05) except in the liver tissues.

#### 6.4.3. Antihypertensive Effects of *G. paraguayense* in Clinical Studies

Inspired by the reported pharmacological activities of *G. paraguayense,* especially the antihypertensive and antioxidant properties of *G. paraguayense* through in vitro, animal, and human studies, a study was designed to conduct a clinical study in Taiwan on people with metabolic syndrome [28]. The effect of the water extract of *G. paraguayense* was studied on blood pressure, blood sugar, lipid profile, and antioxidant enzyme activities of the people divided into control and treatment groups randomly. A dose of 4 g was chosen according to previous clinical and animal studies and was provided for 12 weeks. After the 12 weeks of the administration of the water extract, the SBP, fasting blood glucose, LDL-C, TC, (*p* = 0.08), and TG (*p* = 0.07) levels were decreased in the treatment group as compared to the control (placebo) group [29], while the levels of HDL-C were significantly increased in the treatment group [29]. The activities of the antioxidant enzymes SOD and CAT were also found to be enhanced in the treatment group as compared to the control group [29]. A correlation between antioxidant enzyme activities and blood pressure, FGB, and lipid profiles was observed [29].

### 6.5. Antioxidant Activities of G. paraguayense

The antioxidant activity of *G. paraguayense* is one of the most studied activities, which has been established through in vitro, in vivo, and clinical studies. Antioxidants mainly neutralize free radicals, which are contributors to numerous diseases including cancer, cardiovascular diseases, autoimmune diseases, diabetes, obesity, etc. [30]. The antioxidant activity of *G. paraguayense* is considered to support other pharmacological activities of *G. paraguayense* such as its anticancer, anti-diabetic, and anti-AD activities [31].

#### 6.5.1. In Vitro Antioxidant Activities of *G. paraguayense*

Researchers evaluated the antioxidant activities of *G. paraguayense* extracts in different solvents including water and 50 and 95% ethanol [5]. Before antioxidant activity, the total phenolic and anthocyanin content of all three extracts was estimated as these phytochemicals are important for antioxidant activities. The antioxidant potential of the extracts was studied through radical-scavenging activity, reducing power, and lipid peroxidation in a liposome. The dose-dependent radical-scavenging activities for all extracts were observed through a 2,2-diphenyl-1-picrylhydrazyl (DPPH) assay and a phenazine methosulfate (PMS)–dihydro nicotinamide adenine dinucleotide (NADH)-based method. The highest antioxidant activities were observed in the 50 and 95% ethanol extracts via the DPPH and PMS-NADH methods, respectively. The antioxidant activities of the extract through the reduction of the Fe3+/ferricyanide complex again revealed the dose-dependent reducing activities of all extracts, which were the highest in the case of the 50% ethanolic extract. Lastly, the studies again revealed the dose-dependent antioxidant activities of all the extracts in an assay measuring the inhibition of lipid peroxidation. The study highlighted the antioxidant activity of *G. paraguayense* extracts, which may be further explored to identify the components in the extracts responsible for the antioxidant activities [5].

To analyze the antioxidant activity of another part of the *G. paraguayense*, researchers have studied the antioxidant activity of its stem through different solvents like in previous studies, i.e., water and 50 and 95% ethanol solvents [5,19]. The total phenolic and anthocyanin content of all three extracts of the stem was estimated. The antioxidant activities of the extracts were studied through radical-scavenging activities (through DPPH-, ABTS^+^-, and PMS-NADH-radical-scavenging activities) and the inhibition of lipid peroxidation in a liposome. The highest antioxidant activities were observed in the 50% ethanol extract by the DPPH and ABTS+ methods. The highest antioxidant activities were observed in water extract by superoxide radical scavenging and the inhibition of lipid peroxidation methods. The total TFC and TAC content also supported the highest antioxidant activities of water extract as the TFC and TAC content was highest in the water extract of *G. paraguayense* [19]. Like previous studies, the significant correlation of TPC and TAC with antioxidant activities of extracts such as DPPH-, ABTS-, and superoxide-scavenging activities was observed. The water extract was selected for study for anticancer activities in further cell line experiments.

In another study, to identify the best harvesting time for *G. paraguayense* according to optimal phytochemical content and antioxidant activities, *G. paraguayense* was harvested at different maturity stages (immature, intermediately mature, and mature) and subjected to phytochemical estimation and antioxidant activity [12]. The selected stages were immature (30 days), intermediately mature (between 30 and 60 days), and mature (more than 60 days) to harvest the plant for the preparation of leaf extract in water and were used for analyzing phytochemicals and antioxidant activities. The selected stages were also used for fermentation with different Lactobacillus species *(Lactobacillus plantarum*, *Lactobacillus acidophilus*, and *Lactobacillus paracasei*) to study the effect of fermentation on phytochemical composition and antioxidant activities [12]. Interestingly, in the case of both fermented and non-fermented conditions, the antioxidant activities calculated through DPPH-, ABTS-, and superoxide-scavenging activities were decreased with the maturity levels of the plant and highest for the leaves from the immature plant.

Flavonoids and phenolic content were also found to be higher in immature leaves compared to mature and intermediately mature leaves in the case of both fermented and non-fermented conditions. The fermentation significantly increased the antioxidant activities of the leaves, which are also attributed to the increase in the flavonoid and phenolic acid content due to the fermentation. Among fermentation conditions, fermentation with *L. plantarum* resulted in the highest antioxidant activities and subsequently the highest increase in flavonoid and phenolic acid content. The antioxidant activities were also studied on cell-line-derived normal liver cells (FL83B cell line) of the mouse. The antioxidant activities of the leaf extract on the activities of antioxidant enzymes, including glutathione peroxidase (GPx), glutathione reductase (GR), catalase (CAT), and SOD activities, were studied for both fermented and non-fermented extracts. Like previous experiments, the highest activities for GR, GPx, CAT, and SOD enzymes were observed in the case of extract from the immature stage plant leaves. Similarly, the highest antioxidant activity of immature plant leaves in fermented conditions was observed for *L. plantarum.* The study concluded that the immature stage of plant leaves has the highest antioxidant activities, and fermentation can increase antioxidant activity by increasing flavonoid and phenolic content [12].

Aqueous extracts of leaves partitioned in different fractions were studied to achieve optimal antioxidant activity. Among fractions, ethyl acetate fraction showed the highest and most significant antioxidant activities in DPPH- and ABTS-radical-scavenging assays in comparison with other fractions used in the study, i.e., n-hexane, n-butanol, and water fractions. Further, glycation can cause the formation of advanced glycation end products (AGEs), which can lead to several serious concerns, including diabetes, cardiovascular diseases, cancer, and AD. Antioxidants have the potential to inhibit the glycation process [32]. The ethyl acetate fraction was found to inhibit AGEs and fructosamine formation more potently than other solvent-partitioned fractions. Similarly, extracts of leaves partitioned in different fractions were able to suppress protein carbonyl formation and α-dicarbonyl compound production in a dose-dependent manner. Again, suppression was strongest in the ethyl acetate fraction compared to the other fractions used in the study. Further, the antiglycation effect of ethyl acetate fraction was studied on HepG2 cells. The ethyl acetate fraction was able to suppress carbonyl formation in HepG2, which has been enhanced with the AGE treatment. Similarly, in the comet assay, the ethyl acetate fraction was able to alleviate the DNA damage in HepG2 cells caused by the AGE treatment [16]. The antioxidant and antiglycation activity of ethyl acetate fraction may be helpful in associated diseases such as diabetes; thus, the ethyl acetate fraction was subjected to HPLC to identify the components present in it and was subjected to activity analysis. Among the phytochemicals present in the ethyl acetate fraction, the highest antioxidant and antiglycation activities were exhibited by GA, which is suggested to be the main component responsible for the antioxidant and antiglycation activities of the fraction.

Inspired by the reported pharmacological activities of *G. paraguayense*, especially its antioxidant and hepatoprotective activities, the researchers studied seven isolated flavonoid compounds (four major and three minor compounds) from the methanolic extract of *G. paraguayense* leaves [11]. The isolated compounds were studied for their antioxidant activities through radical-scavenging activities (DPPH and ABTS^+^), reducing power, and the inhibition of lipid peroxidation in a liposome. All seven compounds showed dose-dependent antioxidant activities in all experiments, i.e., DPPH- and ABTS^+^-radical-scavenging activities, reducing power and inhibiting lipid peroxidation [11]. The study suggested that the four major compounds may be further analyzed for their pharmacological activities in future studies.

Considering the potential of leaves from *G. paraguayense* juice as a functional food drink and its macromolecular hydrocolloids for nutraceutical supplementary tablets, the compositional analysis of its leaves and their hydrocolloids was carried out. Extract of leaves in water (LEW) and its 72% ethanol precipitate-containing macromolecules (LEWP) were studied for their antioxidant activities through the scavenging of superoxide anions, DPPH, and ABTS radicals, reducing power, and chelating ability on Fe^2+^ ions. Effective scavenging activities in all experiments in the case of both LEW and LEWP were observed and were higher in LEW for ABTS and superoxide anion scavenging. Conversely, antioxidant activities through the scavenging of DPPH radicals and Fe^2+^ ion chelation were higher in LEWP [6].

#### 6.5.2. In Vivo Antioxidant Activities of *G. paraguayense*

Given the antioxidant activities in different in vitro assays of *G. paraguayense* extract, the in vivo antioxidant activities of *G. paraguayense* were studied. The 50% ethanolic extract that was found to be active in the previous study was prepared and used to study antioxidant activities in animal studies by analyzing enzyme activities in the liver, heart, and brain [5,33]. *tert*-butylhydroperoxide (t-BHP) was used to induce oxidative stress in male Wistar rats, which were grouped into four groups (control, *G. paraguayense* extract alone, t-BHP alone, and *G. paraguayense* extract and t-BHP combined). The rats’ food intake and total body weight did not change significantly between the groups throughout the study. Hepatotoxicity markers such as AST and ALT activities were found to be decreased by the extract treatment which had been increased by the administration of t-BHP [33]. SOD activity was found to be decreased by t-BHP in liver and brain tissues, which had been significantly increased by treatment with the extract in the liver tissues [33]. The extract treatment alone and in combination with t-BHP suppressed MDA levels in the heart tissues. The level of the antioxidant GSH was significantly decreased only in the liver tissues in the t-BHP group as compared to the control. Importantly, the GSH level was not significantly changed in the group that received *G. paraguayense* extract and *G. paraguayense* extract with t-BHP. Similarly, levels of vitamin C and vitamin E were decreased in the tissues in the t-BHP group but were not effected in the treatment group. The study concluded that the antioxidant effects of *G. paraguayense* may be responsible for the protective activities of the liver and brain against t-BHP-induced oxidative stress [33].

In another study, in view of the antioxidant and hepatoprotective activities of *G. paraguayense*, its effects were studied on ethanol- and CCl_4_-induced oxidative stress and liver toxicity in animal models [15]. Sprague–Dawley (SD) rats were randomly divided into five groups in the study, i.e., control (normal saline), only treatment (extract treatment alone), treatment (CCl_4_, ethanol, and extract treatment), negative control (CCl_4_ and ethanol) and positive control (CCl_4_, ethanol, and silymarin) groups. The total antioxidant status (TAS) of serum was significantly increased by the extract treatment, which had been suppressed due to CCl_4_ and ethanol administration. Similarly, the levels of antioxidants such as glutathione (GSH), vitamin C, and vitamin E were also increased in the liver by the extract treatment, which had been suppressed due to CCl_4_ and ethanol-induced toxicity. Additionally, the activities of antioxidant enzymes such as SOD, GPx, CAT, and glutathione S-transferases (GSTs) were suppressed due to CCl_4_ and ethanol-induced toxicity but were later recovered through the extract treatment [15]. The hepatoprotective effects of the extract treatment were also observed in the study (mentioned in the hepatoprotective activity section). Further, the HPLC-based identification of phytochemicals revealed the presence of important flavonoids in the extract, which may be the main reason for the pharmacological properties of the extract.

#### 6.5.3. Antioxidant Activities of *G. paraguayense* in Clinical Studies

Given the role of oxidative stress in patients suffering from hypercholesterolemia and related complications, the role of the consumption of the antioxidant plant *G. paraguayense* on antioxidant status and serum lipid profile was studied [28]. In the study, 100 g of *G. paraguayense* was provided for eight weeks to 18 subjects suffering from hypercholesterolemia. Antioxidants such as ascorbic acid and α-Tocopherol were significantly increased in the blood plasma of the treatment group. Similarly, the activities of antioxidant enzymes such as GPx, GSH, and CAT in erythrocytes were significantly increased in the treatment group. Furthermore, the plasma level of MDA, which is a biomarker of major oxidative stress, was found to be significantly decreased in the treatment group. However, significant changes in the activity of SOD and the levels of serum total cholesterol, triglyceride, low-density lipoprotein cholesterol, and high-density lipoprotein cholesterol were not observed in the study. The study highlighted the antioxidant effect of *G. paraguayense* in patients suffering from hypercholesterolemia and suggested pursuing the further studies with a larger sample size.

### 6.6. Hepatoprotective Activity

Liver disease is one of the major public health problems, causing two million deaths annually worldwide [34]. Liver disease can also increase the risk of a variety of serious diseases in other organs, including chronic kidney disease, diabetes, and heart failure [35,36]. A study was conducted to explore the liver-protective effect of *G. paraguayense* in animal models. The effect of *G. paraguayense* against CCl_4_-induced liver toxicity on SD rats was studied. Three different doses of *G. paraguayense* water extract (50, 150, and 300 mg/kg), a positive control (treatment with silymarin), and a normal control were used in the study [10]. A reduction in the weight of the liver was observed in the CCl_4_-administered group as compared to the control group, which suggested liver injury. The reduction in the liver weight due to CCl_4_ toxicity was recovered in the extract treatment (300 mg/kg) group in the study. Further, the major markers of liver injury, i.e., AST and ALT, were highly increased in the serum of the animals in the CCl_4_-administered group but were significantly suppressed by all the extracts. Similarly, the serum and liver levels of TC and triglycerides (TGs) were also elevated with CCl_4_ administration and significantly suppressed by the extract (300 mg/kg) treatment in the study. The level of MDA that is generated through oxidative lipid peroxidation was highly increased in the CCl_4_ but was significantly suppressed in all treatment groups. The level of GSH and the activities of antioxidant enzymes (GPx, SOD, CAT, and GR) were also suppressed due to the oxidative stress induced by the CCl_4_. The level of GSH and activities of GPx, SOD, CAT, and GR were increased significantly in all treatment groups, which suggests the effective antioxidant-based liver-protective activity of the extract. Oxidative stress inflammation is highly associated with liver damage; thus, the important cytokine TNF-α, which can initiate the inflammatory cascades, was also studied through ELISA. The study revealed that the expression level of TNF- α was suppressed with the extract treatment, which has been increased due to the administration of CCl_4_. Finally, the histopathology visualized the damage to liver tissues through cytoplasmic vacuolization, increased fatty degeneration, and necrosis. All these liver tissue damage-related parameters were improved through the extract treatment in the study, which suggested the effective hepatoprotective activities of *G. paraguayense* extract and the antioxidant and anti-inflammatory properties of extract may be the important contributors to the hepatoprotective activities [10].

In another study, an 80% ethanolic extract of *G. paraguayense* leaves was used to study liver fibrosis [37]. Liver fibrosis mainly occurs due to chronic liver diseases, characterized by the progressive accumulation of the extracellular matrix (ECM), which suppresses liver function and can lead to hepatocellular carcinoma [38]. In liver fibrosis, hepatic stellate cells (HSCs) play a crucial role as the activated HSCs proliferate and excessively produce ECM. The effect of the leaf extract on dimethylnitrosamine (DMN)-induced liver fibrosis was studied in a rat model. Before the animal study, ten traditional Chinese medicines, comprising six medicinal plants (*Graptopetalum paraguayense*, *Phyllanthus urinaria*, *Salvia miltiorrhiza*, *Bupleurum falcatum* L., *Panax pseudoginseng* var. Notoginseng, and *Astragalus membranaceus*) and four formulas were used to study their sensitivity on HSCs [10]. Among all the medicinal herbs and formulas, *G. paraguayense* was found to be most active against HSCs [37].

The animal study was conducted on DMN-induced liver fibrosis in four groups, i.e., control, DMN, DMN + extract treatment, and DMN+ silymarin treatment (positive control) groups. The condition of the animals’ livers was better in the DMN + extract treatment group even when compared with the positive control (DMN+ silymarin-treated group). DMN toxicity caused a reduction in both the liver and body weights of rats in the DMN-treated group, which were increased in both the extract and silymarin treatment groups. In a histopathology analysis, DMN caused large necrosis and inflammatory responses like the infiltration of inflammatory cells and necrosis in different areas. These necrosis and inflammatory effects of DMN were significantly suppressed (30 and 31% reduction in bridging fibrosis and necro-inflammatory responses, respectively) with the extract treatment, which was a better result than that of the positive control. In the DMN treatment rats, the expression of α-SMA, a marker of HSC activation, was found to be enhanced with intense staining in the pericentral area of the liver in the IHC staining experiment. However, the extract treatment suppressed the expression of α-SMA. Further, the TUNEL staining revealed an increase in TUNEL-positive cells around the α-SMA-containing region, implying colocalization in *G. paraguayense*-treated livers. It also suggests that a reduction in the activation of HSCs in fibrotic livers by the extract may be mediated via apoptosis. Additionally, in vitro cell culture of the HSCs, which activated to produce ECM in liver fibrosis, was used to study the effects of the extract. The expression of both SMA and collagen was observed at day 10 and was suppressed with the inclusion of the extract. These results suggest that the extract treatment suppressed the activation of HSCs and the expression of α-SMA and collagen-I. The expressions of known genes in liver damage were studied, such as tissue inhibitors of metalloproteinases (such as Timp1) that can inhibit matrix degradation; transforming growth factor beta-1 (such as Tgfb1), a known inducer of fibrogenesis in the effector cells of hepatic fibrosis that can stimulate adipocyte transformation; and peroxisome proliferator-activated receptor (Pparg) transcription factor, involved in the signaling pathway of lipid metabolism. These expressions, studied through reverse transcription quantitative polymerase chain reaction (RT-qPCR) and microarray methods, revealed that the expression of Timp1 and Tgfb1 was increased in the liver of the DMN-administered group but was suppressed in the extract treatment group. Similarly, the expression of the Pparg transcription factor was enhanced in the extract treatment and reduced in the SMN-administered group, which supports protection against liver damage, as per the literature.

The microarray study revealed that among 256 known liver damage-related discriminator genes, the expression of 64% was recovered significantly after *G. paraguayense* treatment as compared to the SMN group, but Silymarin treatment was able to restore only 9% of those genes. The microarray results suggested a higher potential for extract treatment as compared to the positive control used in the study.

Further, RT-qPCR of five novel marker genes (Btg2, Egr1, Oldr1, Nrg1, and Hmgcs1) was also performed. The results found that a positive correlation was present between the expressions obtained from RT-qPCR and microarray methods. The expression of these genes was altered due to DMN administration recovery with the extract treatment in the RT-qPCR study.

In another study, the effect of methanolic extract of leaves (MEL) was studied through DMN- and CCl_4_-induced hepatic fibrosis on male SD rats. In the case of DMN-induced toxicity, the survival rate, BW, and LW were decreased significantly, which were recovered by the MEL treatment. The spleen weight was increased due to the DMN administration, which indicated the spleen toxicity also recovered with MEL treatment [39]. The survival rate due to DMN toxicity was decreased to 61.1%, which was recovered to more than 94% with the MEL treatment. In a biochemical analysis of serum, prothrombin time and the levels of AST, ALT, and bilirubin were increased due to liver toxicity caused by the DMN. These parameters related to liver toxicity were recovered with MEL treatment. Conversely, the levels of platelets and albumin were suppressed in serum analysis due to DMN also being recovered (enhanced) by the MEL treatment. In the histopathology analysis, hematoxylin–eosin staining revealed liver fibrosis symptoms such as continuous fibrotic septa between the central and portal veins, and periportal and centrilobular deposition of fibers was observed due to DMN toxicity. The suppression of fibril deposition and a significant reduction in collagen levels, through computer-assisted morphometric analysis of liver sections, was observed with the MEL treatment. Hepatic hydroxyproline estimations also revealed similar results; i.e., the collagen content was significantly higher in the DMN groups as compared to the control and MEL treatment group.

In the case of CCl_4_-induced toxicity, survival rate and BW were decreased significantly but were recovered by the MEL treatment. Similarly, spleen splenomegaly was observed due to the administration of CCl_4_, which also recovered with MEL treatment. Like the case of DMN, the biochemical analysis of serum revealed an increase in prothrombin time and the levels of AST, ALT, and bilirubin due to CCl_4_ toxicity. These parameters related to liver toxicity were recovered (suppressed) with MEL treatment. Similarly, the levels of platelets and albumin were increased in the serum analysis due to DMN also being recovered by the MEL treatment. Further, the histopathology analysis revealed a similar result as the liver fibrosis symptoms appeared with CCl_4_ toxicity, like diffuse fatty tissue and micronodular fibrosis around the portal area and the central vein. These fibrosis-related symptoms were significantly suppressed by the MEL treatment. In the staining study, an excessive accumulation of collagen was observed in the CCl_4_-administered group, which was significantly reduced by the MEL treatment. The hepatic hydroxyproline content analysis also revealed similar results, i.e., the collagen content was significantly higher in the CCl_4_-administered group as compared to MEL treatment groups.

The crucial part of liver fibrosis is the activation of HSCs cells that express α-SMA and transform to myofibroblast-like, collagen I-expressing cells. Thus, an analysis was conducted to study the effect of MEL on the activation of HSCs. Primary cultured HSCs were isolated from rats. After 7 days of incubation, the HSCs were transformed into myofibroblast-like cells expressing α-SMA and collagen I. The addition of MEL to HSC culture suppressed the activation and the expression of α-SMA and collagen I. Further, the study also revealed that the MEL treatment induced apoptosis of HSCs as it reduced the viability of HSCs in a dose-dependent manner. However, the viability of normal primary cultured hepatocytes was not suppressed by the MEL treatment at 400 ug/mL. The TUNEL assay revealed the presence of genomic DNA fragmentation as a sign of apoptosis in HSCs with the treatment of MEL, which was missing in the control [39]. In addition, the role of caspase in MEL-induced apoptosis was analyzed. The MEL treatment increased caspase-2, caspase-3, caspase-8, and caspase-9 activities, and inhibitors of these were able to attenuate MEL-induced apoptosis, which suggested the role of the caspase-mediated pathway in the apoptosis of mature HSCs. Further, a WB analysis revealed the suppression of anti-apoptotic proteins (Bcl-2 and Mcl-1) and enhancement in pro-apoptotic molecules, such as Bax and Fas, in the MEL treatment. Kupffer cells are also considered an important contributor to liver inflammation and fibrosis at the initial stages; thus, the effect of MEL on Kupffer cells was also studied. The inflammatory response in terms of the enhanced expression of TNF-α, IL-6, and NO in LPS-induced Kupffer cells of rats was suppressed by the MEL treatment in the study. However, the MEL treatment enhanced IL-10, which may exert an anti-inflammatory effect. Using immunostaining with antibodies in a histopathology analysis, the suppression of TNF- α and IL-6 was also observed in the liver of rats with MEL treatment, which was increased by CCl_4_ toxicity.

Later on, to decipher the molecular mechanism behind the anti-liver-fibrosis activity of *G. paraguayense* extract and 30DE3 fraction in the earlier study, in vitro and in vivo studies were conducted. *G. paraguayense* extracts in various solvents, such as butanol, acetone, 70% methanol, 95% ethanol, 70% ethanol, 50% ethanol, 100% DMSO, and 30% DMSO, were prepared. Based on the inhibition of HSC-T6 cells and the suppression of Aurkb protein, the 30% DMSO extract (30DE) was selected for further studies. 30DE3 fraction was prepared from 30DE, like in the earlier study [20]. The effective inhibition of viability of HSC-T6 and LX-2 cells was observed for the 30DE3 fraction in the MTT assay (Table 2). However, both 30DE and 30DE3 do not have an effect the viability of primary cultured mouse hepatocytes [40]. Profibrogenic cytokine, TGF-β1, which is known to increase the expression of collagen and aurora kinase B (AURKB) and stabilize microtubules and AURKB, was used to stimulate the HSC-T6 and LX-2 cells. In a WB analysis, TGF-β1 enhanced the protein expression of both collagen I and α-SMA. The treatment with 30DE and 30DE3 inhibited the proliferation of both HSC-T6 and LX-2 cells in the presence as well as the absence of TGF-β1 in a time- and dose-dependent manner, which had been enhanced with TGF-β1. Additionally, the protein expressions of collagen I, collagen III, elastin, and α-SMA induced by TGF-β1 were significantly suppressed by 30DE and 30DE3. The SMAD-independent pathway, i.e., the MAPK pathway and SMAD-dependent pathway, is known to be activated by TGF-β1. Thus, the effects of 30DE and 30DE3 were also studied on important proteins of these pathways. In the WB analysis, the suppression of p-P38/P38, p-SMAD2/SMAD2, and p-SMAD3/SMAD3 was observed by 30DE and 30DE3, which had been enhanced in the TGF-β1-treated HSC-T6 cells. 30DE and 30DE3 treatment also suppressed the level of phosphorylated mitogen-activated protein kinase (MEK) in the TGF-β1-treated HSC-T6 cells [40]. The activation of MEK and P38 is known to promote the migration of cells; thus, the effect of 30DE3 on cell migration was also studied through Transwell invasion and wound healing assays. In both assays, 30DE3 effectively inhibited the migration/invasion of HSC-T6 cells in a dose-dependent manner.

Finally, the anti-liver-fibrosis effect of 30DE3 was studied on an animal model using Wistar rats [40]. Liver damage and fibrosis were induced through DEN in the rats. Markers of liver damage, such as ALT, AST, and GGT, were enhanced in the DEN-treated group of animals but were suppressed in the animals of the 30DE3 treatment group and the sorafenib treatment (positive control) group. Similarly, an increase in α-SMA-positive cells (activated HSCs) was observed in the liver of DEN-treated animals, which was suppressed by 30DE3 treatment. The expression of fibrosis-related proteins such as collagen I, collagen III, and α-SMA was also suppressed by the treatment but was increased by DEN in the liver. Finally, the phosphorylation of SMAD2 and p38, which are associated with the fibrosis-related TGF-β1 signaling pathway, were found to be suppressed by the 30DE3 treatment but was significantly increased in the liver of DEN-administered rats. The findings of the study suggested that the anti-liver-fibrosis activity of 30DE3 may be associated with the inhibition of TGF-β1 signaling for the activation of HSCs [40].

In another study, the hepatoprotective and antioxidant effect of *G. paraguayense* was studied on CCl_4_- and ethanol-induced liver toxicity in SD rats. The total antioxidant status (TAS) of serum was significantly increased by the extract treatment, which has been suppressed due to CCl_4_ and ethanol administration. Similarly, the levels of antioxidants such as glutathione (GSH), vitamin C, and vitamin E were also increased in the liver by the extract treatment, which had been suppressed due to CCl_4_- and ethanol-induced toxicity. Additionally, the activities of liver enzymes such as LDH, AST, and ALT in plasma were increased due to ethanol and CCl_4_ toxicity, indicating hepatic damage. However, the extract treatment significantly suppressed the levels of LDH, AST, and ALT in plasma, indicating the protective effects of the extract [15]. Further, histopathological analyses investigated the protective effects of the extract; the accumulation of hepatic triglyceride, increased vacuole formation, deformed liver architecture with fatty lesions due to intensive fatty infiltration, and signs of necrosis were suppressed due to extract treatment. Further, antioxidant-related parameters also improved due to the extract treatment (mentioned in Section 6.5.2). The HPLC-based identification of phytochemicals revealed the presence of important flavonoids in the extract, which may be the main reason for the pharmacological properties of the extract [15].

In a recent study, the liver-protective effect of *G. paraguayense* was analyzed in Methylglyoxal (MGL)-induced liver toxicity in an animal model [41]. Water extract (WEGP) prepared through ultrasonication-based extract at room temperature was used for the treatment (in three doses, i.e., 50, 250, and 500 mg/kg) of MGL-induced liver toxicity in male SD mice. In the study, the serum activity of ALT was increased by 47% due to the MGL, which was reduced by the WEGP by 14% compared to the MGL group. Similarly, the serum activity of serum ALP was significantly increased in the MGL group, which was significantly suppressed by the WEGP treatment. Conversely, the level of AST was not significantly changed in any group in the study. The serum levels of TC and TG levels were found to be decreased significantly in a dose-dependent manner with WEGP treatment, which were also increased significantly in the MGL treatment group. Similarly, the serum levels of LDL-c and LDL-c/HDL-c were found to be decreased significantly in the WEGP treatment groups as compared with the MGL treatment group. Higher levels of LDL-c and LDL-c/HDL-c are associated with a high risk of cardiovascular disease (CVD). However, the serum level of HDL-c was not changed in any group of animals in the study [41]. MGL is known to exert oxidative stress by suppressing the antioxidant status and activities of antioxidant enzymes. In line with the literature, the activities of antioxidant enzymes including GR, GPx, and CAT were significantly suppressed due to MGL administration, which were restored by the WEGP treatment in the study. Inflammation is an important factor in liver damage; thus, the serum levels of the inflammatory cytokines TNF-α and IL-6 were also studied. The serum levels of both inflammatory cytokines were significantly elevated with the MGL treatment, which were significantly suppressed by WEGP in a dose-dependent manner, and the suppression by the high dose of WEGP was higher than in the positive control (N-acetyl-L-cysteine). In a WB analysis, the protein expression of TGF-1 was found to be increased in the liver of MGL-treated animals, which was suppressed by the WEGP in an almost dose-dependent manner in the study. The suppression of TGF-1 with *G. paraguayense* was also observed in the earlier studies, which support the hepatoprotective properties of *G. paraguayense* through TGF-I-related signaling disruption. The content of MGL in the liver and serum was also analyzed in the study as MGL levels are associated with diabetes and the formation of toxic reactive glycation products. In the study, a high concentration of MGL in both the liver and serum was observed, which was suppressed by the WEGP treatment [41].

The effect of *G. paraguayense* on intestinal inflammation and microflora was studied through an animal study of male C57BL/6J fed on a high-fructose diet. Shortening of intestinal length is a symptom of inflammatory bowel disease; the length of the large intestine was studied, which was found to be significantly decreased in animals consuming a high-fructose diet (HFD) [41]. However, the WEGP treatment significantly increased the length of the large intestine in comparison with the HFD group. Similarly, the level of inflammatory cytokine TNF- was also suppressed by the WEGP treatment, which was elevated by the HFD. Intestinal microflora dysbiosis was indicated in the HFD group as the ratio of Bacteroidetes to Firmicutes was found to be decreased compared to the control group. However, the ratio of Bacteroidetes to Firmicutes was restored by WEGP treatment, which was similar to the control in the high-dose WEGP group [41]. Further, long-term high intake of fructose may cause a reduction in tight-junction proteins, such as occludin and claudin-1. A reduction in these proteins can increase intestinal permeability, which may be associated with chronic inflammation of the intestine. In comparison with the control group, the levels of both occludin and claudin-1 were significantly reduced by HFD, which were effectively increased by the WEGP treatment [41].

### 6.7. Anti-Diabetic Activity

Advanced glycation end products (AGEPs) are known to create oxidative stress, which generates pro-inflammatory cytokines [42,43]. AGEPs are known to damage the pancreas and cause hyperglycemia through their receptors. Major AGEPs such as carboxymethyllysine (CBML) can increase MDA levels and can also be responsible for hyperglycemia.

Natural products have shown anti-diabetic activity along with antioxidant activities in different studies [44,45]. The anti-diabetic activity of *G. paraguayense* was studied through the protective activity of the ethanolic extract of leaves from *G. paraguayense* (EEL) on CBML-induced pancreatic damage in animal models. Ethanolic extract was prepared through the extraction of freeze-dried and powdered leaves of *G. paraguayense* with 95% ethanol. Five groups were used in the study, which were control (CG), CBML (CBMLG), CBML + resveratrol (RESG), CBML + gallic acid (GAG), and CBML + EEL (EELG). An oral glucose tolerance test (OGTT) before CBML induction revealed a significant reduction in glucose by resveratrol, gallic acid, and EEL administration in the mice. Importantly, the level of insulin was also found to be significantly suppressed by resveratrol, gallic acid, and EEL administration, which suggested that insulin sensitivity was increased with treatment [46]. After the 12-week treatment, the inhibition of insulin and elevation in glucose were observed in the CBMLG. However, all three treatment groups recovered insulin levels and reduced glucose levels in the study [46].

Pancreatic damage was observed via IHC staining analysis in the CBMLG after a 12-week experiment. The number/area of islet cells and insulin levels were decreased in the CBMLG, which were remarkably restored in all treatment groups in the study. A mechanistic analysis was also conducted to decipher the possible mechanisms behind the anti-diabetic treatment. The expression of PPAR-γ and PDX-1 in the pancreas is positively associated with insulin synthesis, and its inhibition is associated with diabetes. The expression of both of these was found to be suppressed in the CBMLG, which was recovered in all three treatment groups. Conversely, C/EBPβ, which suppresses the transcription of the precursor of insulin was elevated in the CBMLG, which was suppressed in all three treatment groups. The antioxidant status of the pancreas was also studied, and the expressions of p-NRF2 and GCL were found to be significantly increased in all three treatment groups compared to the CBMLG.

In the CBMLG, the level of GSH was reduced, and the amount of MDA, which is an indicator of lipid oxidation, was increased. However, the levels of both GSH and MDA were significantly recovered in all three treatment groups in the study [46].

In the liver, the levels of GSH, GCL, p-Nrf2, and MDA were also exhibited a similar pattern in the pancreas; i.e., the levels of GSH, GCL, p-Nrf2, were found to be significantly increased in all three treatment groups compared to the CBMLG and vice versa for MDA.

The effect of treatment was also studied on insulin sensitivity in the liver and muscles of the mice. The activation of AKT is crucial for glucose uptake in cells, i.e., the phosphorylation of AKT; an indicator of glucose uptake, 2-[N-(7-nitrobenz-2-oxa-1,3-diazol-4-yl)amino]-2-deoxy-D-glucose (2-NBDG), which was inhibited by the CBML, was found to be enhanced by all three treatments [46].

### 6.8. Tyrosinase Inhibitory Activity

Tyrosinase is a multifunctional enzyme that involves browning in plants, which may change their color, flavor, and nutritive value [47]. It also involves melanin synthesis, which is responsible for pigmentation. Tyrosinase’s multiple functions are responsible for the importance of tyrosinase inhibitors (TIs) in different industries such as food, agriculture, cosmetics, and medicine. TIs contribute to supporting the flavor, color, and nutritional values of food and agriculture products. In cosmetics and medicine, its main role is in skin whitening and pigmentation-related issues, respectively. Natural TIs are preferred over synthetic ones as they are used in medicine, cosmetics, and food and are considered to be safer [48]. The tyrosinase inhibitory activity of *G. paraguayense* was studied through mushroom tyrosinase on Dopa oxidation inhibition, and kojic acid was used as the reference (known TI). Various *G. paraguayense* extracts were prepared using water and 50 and 95% ethanol as solvents [49]. In an enzymatic assay of mushroom tyrosinase, the highest activity was shown by kojic acid. However, all the *G. paraguayense* extracts showed effective dose-dependent tyrosinase inhibitions. Among the extracts, the 95% ethanolic extract had the highest tyrosinase inhibition activity, followed by the 50% ethanol and water extracts. Further, Lineweaver–Burk plots of enzyme inhibition indicated a mixed type of inhibition pattern in three extracts [49]. The study suggested the potential of *G. paraguayense* in the cosmetics, food, and medicine industries. However, further research is required to study the effect of *G. paraguayense* in vitro and in vivo melanin formation and to identify the potential constituent from *G. paraguayense* that is responsible for the activities.

### 6.9. Antiviral Activity

The antiviral activity of *G. paraguayense* leaf extract was studied on herpes simplex virus type 1 (HSV1) and type 2 (HSV2). HSV can cause unnoticeable signs or symptoms from orofacial and genital lesions to deadly encephalitis. Genital HSV disease can also induce cervical cancer and increase the chances of transmission of human immune deficiency virus. HSV also suppresses immunity and increases the chances of co-infection with pathogenic bacteria, which may create life-threatening complications. The issue of drug resistance is also worsening the effectiveness of HSV treatment as drug resistance and toxicity against standard drugs (acyclovir) and their analogs have been reported. Thus, the overall scenario demands new therapeutics with different mechanisms of action that can overcome the situation. The recent literature has supported some phytochemicals as anti-HSV agents, which may have lesser toxicity and chances of drug resistance in HSV treatment. The leaf extract of *G. paraguayense* was prepared in 75% methanol to study its anti-HSV effects in two mutants (HSV-1 strain DD and HSV-2 strain PU) and two wild strains of HSV (HSV-1 strain Victoria and HSV-2 strain Bja) with acyclovir as the reference drug [50]. Before the anti-HSV study, the cytotoxicity of the extract was analyzed against the Vero and RD cell lines derived from the green monkey kidney and human rhabdomyosarcoma, respectively. Concentrations of up to 1 mg/mL of extract was found to be safe in both observations of the morphology through a microscope and an MTT assay. In both analyses, i.e., microscopic observation and the MTT assay, the cytotoxicity of the extract in terms of its maximal nontoxic concentration (MNC) and EC_50_ were less in comparison with the positive control drug used in the study. The antiviral activity of the extract was studied through the inhibition of HSV reproduction in Vero cells. The antiviral activities of the extract at MNC were different for different HSV strains, which suggested the extract may be specific and selective against the HSV. The activities of the extract at MNC were highest and lowest for HSV-1 (strain Victoria) and HSV-2 (strain PU), respectively (Table 2). The study suggested that *G. paraguayense* may be used along with approved anti-HSV-1 drugs after analyzing its interaction with the drug. Further anti-HSV mechanisms of *G. paraguayense* may be studied in future studies [50].

Hepatitis B virus can be responsible for acute and chronic hepatitis, which may lead to serious liver cirrhosis or hepatocellular carcinoma. A large number of people are infected with HBV, and it has been estimated that approximately 254 million people are living with chronic hepatitis B worldwide [51]. The current treatment strategy is the long-term suppression of virus replication. Drug resistance and side effects from long-term use are issues associated with HBV treatment. Plant-based antiviral drugs are considered to be more effective in terms of low toxicity and drug resistance [52,53]. The anti-HBV activities of *G. paraguayense* were studied on liver cell lines that express HBV genes, i.e., Hep3B/T2 and 1.3ES2 cell lines [54]. These studies suggested that PGC-1α, a regulator of lipogenesis and gluconeogenesis, strongly coactivates HBV gene expression in the liver. The effects of 30DE3 on Hep3B/T2 resulted in the suppression of (*G6Pase*) and phosphoenol pyruvate carboxykinase (*PEPCK*) gene expression, which was enhanced by 8-Bromo-cAMP and dexamethasone (8-BCAMP/DEX). Similarly, 30DE3 suppressed the gene and protein expressions of PGC-1α, which were enhanced by the 8-BCAMP/DEX. Additionally, 30DE3 also suppressed the protein levels of FOXO1 and HNF4α, which are gluconeogenic. In a luciferase promoter activity assay, 30DE3 suppressed hepatitis B viral core promoter activity, which was enhanced by 8-BCAMP/DEX. The dose-dependent inhibition of HBV surface antigen (HBsAg) secretion from Hep3B/T2 cells was observed with the 30DE3 treatment. The 30DE3 treatment in the 1.3ES2 cell line, which comprises the entire HBV genome, was able to suppress the gene expressions of G6Pase, PEPCK, and PGC-1α. Similarly, the 30DE3 treatment suppressed HBV mRNA, core protein levels, and viral replication in 1.3ES2 cells. The study suggested that 30DE3 target gluconeogenesis inhibits HBV [54].

### 6.10. Antibacterial Activity

The antibacterial activities of *G. paraguayense* leaf extract were studied against both Gram-positive—i.e., *Staphylococcus aureus* (SA), methicillin-resistant SA (MRSA), *Enterococcus faecalis* (EF), and *Streptococcus pyogenes* (SP)—and Gram-negative—i.e., *Pseudomonas aeruginosa* (PA) and *Escherichia coli* (EC)—bacterial strains. The Broth microdilution test was used to study the inhibitory activities of the extract on all the selected bacterial strains [50]. Anti-biofilm activity was studied against the MRSA through the biofilm formation assay. The results showed higher antibacterial activities for Gram-positive bacterial strains as compared to Gram-negative strains (Table 2). Further, effective anti-biofilm activity of the extract was observed against MRSA, which was 90% at 2.5 mg/mL. The study suggested the need for the identification of components of the extract responsible for the antibacterial and anti-biofilm activities to achieve optimal activity [50].

### 6.11. Anti-Inflammatory Activity

After achieving effective antioxidant and anticancer activities of the extract of leaves in water (LEW) and its 72% ethanol precipitate-containing macromolecules (LEWP), a preliminary study evaluating the potential for anti-inflammatory activity in brain cells was conducted on both LEW and LEWP. The effect of LEW and LEWP pretreatment was studied on LPS-induced inflammation in the murine microglia cell (BV-2) line [6]. In the study, LEW pretreatment was able to strongly suppress the expression of both inflammatory TNF-α and IL-6 markers, which suggested the effective antioxidant potential of LEW. Conversely, in the case of LEWP, the expression of both inflammatory markers (TNF-α, and IL-6) was increased. The study suggested that LEW as an anti-inflammatory candidate may pursued in further in vivo studies.

Chronic inflammation is an important risk factor for metabolic syndrome (MS) [55,56]. Recently, *G. paraguayense* has been shown to have positive effects on MS patients through regulating blood glucose, blood lipids, and blood pressure. Considering the association of inflammation and MS, the study was designed to analyze the effect of *G. paraguayense* on chronic inflammation in MS subjects. The extract of freeze-dried and powdered leaves of *G. paraguayense* was prepared in water, and 4 g/day of this water extract was consumed after a meal over 12 weeks of treatment [8]. After the 12 weeks of treatment, parameters related to inflammation such as CRP, TNF-*α*, and IL-6 were significantly suppressed in comparison with the baseline. Similarly, antioxidant enzyme activities including SOD and CAT were increased in comparison with the baseline. Similar results were observed in comparison with the placebo group, i.e., CRP, TNF-*α*, and IL-6, which were significantly suppressed, and SOD and CAT activities were significantly increased with the extract treatment. It is the first clinical study that highlighted the anti-inflammatory activities of *G. paraguayense*. However, further studies may be required to confirm the beneficial effects of *G. paraguayense* as a supplement and/or treatment in MS patients [8].

The anti-inflammatory activity of *G. paraguayense* was found to be protective of organs such as the liver and brain, which inspired researchers to study the effects of *G. paraguayense* extract on airway inflammation in mice models [57]. Like earlier studies, ethanol (95%) was used to prepare an extract of powdered, freeze-dried *G. paraguayense* leaves. Ovalbumin-induced allergic airway inflammation in a mice model was used for study. Ovalbumin caused the infiltration of inflammatory cells into lung tissues. A histopathology of lung tissues and analysis of bronchoalveolar lavage fluid (BALF) revealed inflammatory cells in the peribronchial and perivascular areas, which were reduced with extract treatment and gallic acid treatment. A cell count analysis revealed that the increases in the number of total cells, eosinophils, lymphocytes, and neutrophils were due to ovalbumin. In BALF, the levels of cytokines of Th2 cells—IL-4, IL-5, and IL-13—were elevated by ovalbumin induction and suppressed in the extract and gallic acid treatment groups. Further, a flow cytometry analysis revealed a high abundance of T cells (CD4 and CD8) in the BALF of ovalbumin groups of mice as compared to the control group, while both the extract- and gallic acid-treated mice recovered with an increase in T cells in the BALF.

The overproduction of mucus was observed in the ovalbumin group of mice through the PAS staining of lung sections. The overproduction of mucus was also suppressed in the both extract- and gallic acid-treated groups.

The immunohistochemistry of the lungs revealed the activation of NRF2 in both the extract and gallic acid treatment groups, which also increased the gene and protein expressions of its downstream enzymes such as GCL and GSTs, which support the antioxidant effects of the extract. Additionally, the level of GSH was increased, and the generation of TBARS was inhibited in the extract and gallic acid treatment groups. The increase in the GSH suggested an improvement in oxidative stress, and the suppression of TBARS indicated the inhibition of peroxidation of lipids.

The inhibition of eosinophil, lymphocyte, and neutrophil recruitment into the lungs was observed in the extract and gallic acid treatment groups, which may be due to the inhibition of intercellular adhesion molecule 1 (ICAM-1) and vascular cell adhesion molecule 1 (VCAM-1). A gene expression analysis through RT-qPCR showed that the enhancement in the expression of *ICAM-1* and *VCAM-1* was inhibited in both the extract and gallic acid treatment groups.

Immunoglobulin E (IgE) plays a key role in allergic reactions; in the study, ovalbumin-specific IgE levels in serum were suppressed in both the extract and gallic acid treatment groups, which were increased in the ovalbumin group. Similarly, the serum level of IL-4 was also suppressed in both the extract and gallic acid treatment groups. The role of Th1 and Th2 transcription factors T-bet and GATA3, respectively, was also studied through WB analysis. The expression of T-bet was unchanged in the study; however, the expression of GATA3 in CD4 T cells and blood monocytes of the ovalbumin-induced mice was suppressed by the extract and gallic acid treatments, which was elevated by the ovalbumin induction. These findings suggest the inhibition of Th2 signaling in the anti-allergic activity of both extract and gallic acid [57].

**Table 2:** Pharmacological activities of *G. paraguayense* in in vitro studies.

Sr. No.	Activity	Plant Part/Extract Type	Method	Result	Ref.
1.	Anticancer activity	Stem extract aqueous solution	Anti-proliferative effect on HepG2 cells	HepG2 cells ↓	[19]
			Cell cycle and apoptosis study by flow cytometry	G0/G1 ↑ and apoptotic cells ↑	
		Leaf extract in 30% DMSO	Anti-proliferative effect on Huh7 and Mahlavu cells	The IC_50_ values were 500 and 250 μg/mL for Huh7 and Mahlavu cells, respectively	[20]
		Fraction of leaf extract in 30% DMSO (30DE3)	Anti-proliferative effect on Huh7, PLC5, and Mahlavu cells	The IC_50_ values were 50, 37.5, and 75 μg/mL, for Huh7, Mahlavu, and PLC5 cells, respectively	
			WB		
			ROS was measured through hydroethidine fluorescence for detecting the intracellular level of superoxide production	The production of superoxide↓ and intracellular peroxide ↓	
		Leaf extract in 50% ethanol (62.5–500 μg/mL)	Viability of A375.S2 cells (melanoma cells) and caspase-3 activity by flow cytometry and stained with PI	Viability of the cells ↓ (IC_50_ 250 μg/mL), apoptotic cells ↑	[21]
		Leaf extract in 50% ethanol (250 μg/mL)	DAPI staining, DNA electrophoresis, and flow cytometry for studying DNA damage, apoptosis, and caspase 3 activity	Chromatin condensation ↑, caspase-3 activity ↑	
			Protein expression through WB	Cyclin A ↓, Cyclin B ↓, CDC2 ↓, CDC25c ↓, CHK1 ↑, CHK2 ↑, Weel ↑, p21 ↑, p53 ↑, SOD, catalase, and AIF ↑, Bax ↑, caspase-9 ↑, Bcl2 ↓	
			Effect on calcium signaling	Intracellular levels of Ca^2+^ ↑, GRP78 ↑, GADD153 ↑, and caspase-7 ↑	
			Mitochondria-dependent apoptotic signals in A375	MMP (ΔΨm) in A375.S2 cells with an early time period of (0.5–2 h) exposure. In addition, the levels of Bax, caspase-9, and AIF in cells were stimulated after GE50 treatment, but the level of Bcl-2 was attenuated in cells	
		500 μg/mL	GADD153, Endo G, cytochrome c, and AIF nuclear translocation in A375.S2 cells through Confocal microscopy	Nuclear translocation of GADD153↑, Endo G ↑, and AIF ↑; Release of cytochrome c↑ from mitochondria to cytosol.	
		250 μg/mL	Activity of MDA, SOD, and catalase studied through spectrophotometry-based methods.	GPx ↑, SOD ↑, and CAT ↑	
		250 μg/mL	ROS production and redox status and 2,7-dichlorodihydrofluorescein diacetate (10 μM), Indo-1-AM (2.5 μg/mL), and 3,3′-dihexyloxacarbocyanine iodide (500 nM) dye were used to determine ROS, Ca^2+^, and MMP	ROS ↓, MMP ↓, and Ca^2+^ ↑	
		Extract of leaves in water (LEW) and its precipitate (LEWP)	Caco-2 cells studied through MTT assay	Caco-2 cells ↓ IC_50_: values were >1 and 0.12 mg/mL for LEW and LEWP, respectively	[6]
2	Anti-Alzheimer disease activity	10, 30, and 50 μg/mL of 30DE3	ELISA for studying Aβ 1-40 and Aβ 1-42 in SH-SY5Y-APP_695_ cells	Aβ 1-40 and Aβ 1-42 secretion ↓	[9]
		5 μg/mL of 30DE3	Microarray L1000 expression profiling and gene set enrichment analysis (GSEA) of HT29 cells	Significant genes were enriched in AD, HD, and AMPK signaling pathways	
		5 μg/mL of 30DE3	Studying the phosphorylation of AMPK in glial U87 cells through WB	pAMPK/AMPK ↑	
		5, 25, and 50 μg/mL of 30DE3	Glial (U87) and neuronal (SH-SY5Y-APP_695_) cells and microtubule-associated protein 1A/1B-light chain 3 (LC3) through WB for studying the autophagy	LC3-II ↑	
			WB on SH-SY5Y-APP_695_ cells	p62 ↓	
		5, 10, 20, and 50 μg/mL of 30DE3	Neurons differentiated from human-induced pluripotent stem cells from AD patients	Aβ 1-40 and Aβ 1-42 secretion ↓	[24]
			Tau phosphorylation in AD-iNs, measured using Western blotting	Tau phosphorylation ↓ at Ser214	
3	Antihypertensive activity	Extract of leaves in water and 50% and 95% ethanol	FAPGG substrate-based assay for ACE inhibition	IC_50_ values were 46.8 ± 2.5, 19.6 ± 2.5, and 13.7 ± 2.0c, for water, 50% ethanol, and 95% ethanol, respectively	[7]
4	Antioxidant activity	Extract of leaves in water and 50% and 95% ethanol	Superoxide-radical-scavenging activity	IC_50_ values were 0.27 ± 0.01, 1.07 ± 0.24, and 0.19 ± 0.01 for water, 50 ethanol, and 95% ethanol extracts, respectively	[5]
			DPPH-radical-scavenging activity	IC_50_ values were 1.65 ± 0.08, 0.29 ± 0.05, and 1.44 ± 0.16 for water, 50 ethanol, and 95% ethanol extracts, respectively	
			Lipid peroxidation inhibition	IC_50_ values were 1.29 ± 0.20, 1.49 ± 0.17, and 1.34 ± 0.20 for water, 50 ethanol, and 95% ethanol extracts, respectively	
		Extract of stem in water and 50% and 95% ethanol	Superoxide-radical-scavenging activity	IC_50_ values were 0.28 ± 0.01, 2.15 ± 0.19, and 3.82 ± 0.25 for water, 50 ethanol, and 95% ethanol extracts, respectively	[19]
			DPPH-radical-scavenging activity	IC_50_ values were 0.35 ± 0.02, 0.32 ± 0.01, and 0.51 ± 0.02 for water, 50 ethanol, and 95% ethanol extracts, respectively	
			ABTS^+^	IC_50_ values were 0.87 ± 0.01, 0.76 ± 0.00, and 1.71 ± 0.06 for water, 50 ethanol, and 95% ethanol extracts, respectively	
			Lipid peroxidation inhibition	IC_50_ values were 0.68 ± 0.02, 1.12 ± 0.03, and 0.67 ± 0.01 for water, 50 ethanol, and 95% ethanol extracts, respectively	
		Leaf extract (from immature, intermediately mature, and mature plants)	GPx, GR, CAT, and SOD enzyme activities on normal liver cells (FL83B cell line)	Immature leaf extract: GPx, GR, CAT, and SOD enzyme activities were 4.1 ± 0.3, 4.7 ± 0.1, 2.2 ± 0.2, and 1.4 ± 0.1, respectively; Intermediately mature leaf extract: GPx, GR, CAT, and SOD enzyme activities were 3.9 ± 0.3, 4.0 ± 0.2, 2.0 ± 0.2, and 1.3 ± 0.1, respectively; Mature leaf extract: GPx, GR, CAT, and SOD enzyme activities were 2.7 ± 0.4, 4.2 ± 0.3, 2.1 ± 0.3, and 1.0 ± 0.1, respectively.	[12]
		Leaf juice from immature plants fermented through La, Lpl, and Lpr		GPx, GR, CAT, and SOD enzyme activities were 4.5 ± 0.1, 5.1 ± 0.2, 2.3 ± 0.2, and 1.9 ± 0.1, respectively, for La fermented leaf juice; GPx, GR, CAT, and SOD enzyme activities were 5.0 ± 0.2, 5.3 ± 0.1, 2.9 ± 0.2, and 2.1 ± 0.1, respectively, for Lpl fermented leaf juice; GPx, GR, CAT, and SOD enzyme activities were 4.1 ± 0.2, 4.9 ± 0.3, 2.3 ± 0.1, and 1.7 ± 0.1, respectively, for Lpr fermented leaf juice	
		Aqueous extract of leaves partitioned in n-hexane, ethyl acetate, n-butanol, and water fractions and compounds isolated from ethyl acetate fraction (**C1**–**C6**)	DPPH- and ABTS-scavenging assays	Among the fractions, ethyl acetate fraction showed the highest DPPH-scavenging activity, and for compounds **C1**–**C5**, the DPPH-scavenging activities were 86.88 ± 0.68, 40.18 ± 1.57, 11.57 ± 3.04, 43.19 ± 1.37, and 9.65 ± 1.79%, respectively	[16]
				Ethyl acetate fraction showed the highest ABTS-scavenging activity	
			Antiglycation activity fluorescence intensities were measured using a spectrofluorometer	Among the fractions, ethyl acetate fraction showed the highest glycation inhibition activity, and for compounds **C1**–**C5**, the glycation inhibition activities were 68.11 ± 4.24, 44.82 ± 6.35, 14.98 ± 3.46, 32.97 ± 4.91, and 19.48 ± 6.13, respectively	
		Compounds (**C6**–**C12**) isolated from methanolic leave extract	DPPH	IC_50_ values for compounds **C6**–**C12** were 7.88 ± 0.57, 144.93 ± 3.56, 8.40 ± 0.06, 108.47 ± 6.94, 11.06 ± 0.34, 351.89 ± 17.53, and 19.18 ± 0.91 μM, respectively	[11]
			ABTS	IC_50_ values for compounds **C6**–**C12** were 5.69 ± 0.31, 96.81 ± 2.40, 9.22 ± 0.67, 152.21 ± 12.16, 14.50 ± 1.24, 277.44 ± 10.01, and 15.28 ± 0.06 μM, respectively	
			Lipid peroxidation inhibition	IC_50_ values for compounds **C6**–**C12** were 10.76 ± 0.10, 9.10 ± 0.17, 46.54 ± 1.01, 17.45 ± 0.18, 1.67 ± 0.01, 330.64 ± 12.63, and 2.96 ± 0.06 μM, respectively	
		Extract of leaves in water (LEW) and its precipitate (LEWP)	DPPH	SC_50_ values were 4.14 and 1.45 mg/mL for LEW and LEWP, respectively	[6]
			ABTS	SC_50_ values were 0.30 and 0.62 mg/mL for LEW and LEWP, respectively	
			Superoxide anion scavenging	SC_50_ values were 0.18 and 0.37 mg/mL for LEW and LEWP, respectively	
5.	Anti-inflammatory activity	Extract of leaves in water (LEW) and its precipitate (LEWP), 0.125–0.50 mg/mL	LPS-induced BV-2 cells and ELISA for studying their expression	TNF-α ↓ and IL-6 ↓ (LEW treatment) TNF-α ↑ and IL-6 ↑ (LEWP treatment)	[6]
6.	Tyrosinase inhibitory activity	Extract in water and 50% and 95% ethanol	Mushroom tyrosinase on Dopa oxidation inhibition	IC_50_ values were 0.80 ± 0.02, 1.14 ± 0.05, and 2.83 ± 0.05 mg/mL for 95% ethanol, 50% ethanol, and water extracts, respectively	[49]
7.	Antibacterial activity	Methanolic extract of leaves	The BMD assay for SA, EF, EC, PA, SP, and MRSA	MIC values were 2.5, 5, >5, >5, 2.5, and 5 mg/mL for SA, EF, EC, PA, SP, and MRSA, respectively	[50]
			Biofilm formation assay for MRSA	MBIC_50_ was 1.6 mg/mL	
8.	Antiviral activity	Methanolic extract of leaves	Cytopathic effect reduction assay	Protection values were 97.5, 65.5, 25.5, and 13% for HSV-1 strain Victoria, HSV-1 strain DD, HSV-2 strain Bja, and HSV-2 strain PU, respectively	[50]
		30DE3	RT-qPCR-based expression analysis on Hep3B/T2	*PGC-1α* ↓, *G6Pase* ↓, and *PEPCK* ↓	[54]
			WB	PGC-1α ↓, HNF-4α ↓, FOXO1 ↓	
			Luciferase promoter activity assay	HBV core promoter activity ↓	
			ELISA	HBV surface antigen (HBsAg) secretion and wild-type HBV DNA	
			1.3ES2 RT-qPCR	*PGC-1α* ↓, *G6Pase* ↓, *PEPCK* ↓, HBV mRNA ↓, wild-type HBV DNA ↓	
			1.3ES2 WB	HBV core protein levels ↓	

BMD: broth microdilution assay; C1: Gallic acid; C2: Isoquercitrin-6-(3-hydroxy-3-methylglutarate); C3: Astragalin-6-(3-hydroxy-3-methylglutarate), C4: Isoquercitrin -2-acetyl-6-(3-hydroxy-3-methylglutarate); C5: Astragalin-2-acetyl-6(3-hydroxy-3-methylglutarate); C6: quercetin 3-O-[6″-(3-hydroxyl-3-methylglutaroyl)]-β-d-glucopyranoside; C7: kampferol 3-O-[6″-(3-hydroxyl-3-methylglutaroyl)]-β-d-glucopyranoside; C8: quercetin 3-O-[6″-(3-hydroxyl-3-methylglutaroyl)-2″-acetyl]-β-d-glucopyranoside; C9: kampferol 3-O-[6″-(3-hydroxyl-3-methylglutaroyl)-2″-acetyl]-β-dglucopyranoside; C10: isoquercetin; C11: kaempferol 3-O-β-d-glucopyranoside; C12: kaempferol; EF: *Enterococcus faecalis*; EC: *Escherichia coli*; *L. acidophilus*, (*L. plantarum* BCRC 10357 fermentation), *L. paracasei* BCRC 14023 fermentation; MBIC_50_: minimum biofilm inhibition concentration 50%; MIC: minimal inhibitory concentration; MRSA: methicillin-resistant *S. aureus*; SA: *Staphylococcus aureus*; PA: *Pseudomonas aeruginosa*; SP: *Streptococcus pyogenes*; GPx: glutathione peroxidase; SC_50_: 50%-scavenging concentrations; SOD: superoxide dismutase; CAT: catalase; ROS: reactive oxygen species; MMP: mitochondrial membrane permeabilization; WB: Western blot; ↑: upregulation; ↓: downregulation/inhibition.

**Table 3:** Pharmacological activities of *G. paraguayense* in in vivo, ex vivo, and human studies.

Sr. No.	Activity	Material and Dose	Model	Method	Results	Reference
1	Anticancer activity	0.6 and 1.8 g/rat of lyophilized *G. paraguayense* powder and 0.036 g/rat of 30DE3 powder per day for 3 weeks	Male Wistar albino rats; disease induced through the carcinogen DEN	Collagen content in liver, measured by levels of hepatic hydroxyproline	Collagen content ↓ (in all treatments)	[20]
				Tumor burden histopathology	Number of tumors ↓ (in all treatments)	
2	Anti-AD activity	30DE3 powder, 300 mg/kg/day	APPswe/PS1dE9 (APP/PS1) double-transgenic mice	Thioflavin-S (ThS) fluorescent staining of brain sections	Deposition of Aβ ↓ (in the cerebral hemisphere)	[9]
				ELISA analysis of cerebral cortex tissues	Soluble and in soluble Aβ1-40 levels ↓	
				WB analysis	pAMPK ↑ and AMPK ↑	
		0, 20, and 40 μg/mL of 30DE3.	Transgenic *Caenorhabditis elegans* carrying GFP::LGG-1	GFP fluorescence-based method	GFP::LGG-1 ↑	
					Nuclear translocation of HLH-30/TFEB ↑	
3	Anti-aging activity	20 μg/mL of 30DE3	Transgenic *Caenorhabditis elegans* carrying 35 polyglutamine repeats (Q35)	Thrashing assay	Mobility ↑	[9]
			Wild-type *Caenorhabditis elegans*	Lifespan analysis	Lifespan ↑	
			daf-16 null mutant *Caenorhabditis elegans*	Lifespan analysis	Lifespan ↑	
4.	Antihypertensive activity	Extract of leaves in 50% ethanol, 2.5 g/kg BW daily for four consecutive weeks	Spontaneously hypertensive rats (SHRs) and age-matched normotensive WKY rats	SBP, DBP, and MBP were observed	SBP ↓, DBP ↓ and MBP ↓, normalized with treatment	[27]
				FAPGG substrate-based assay for ACE inhibition	ACE activity ↓ (normalized in plasma, kidneys, and lungs)	
				2,2′-azinobis-(3-ethyl benzothiazoline-6-sulfonic acid (ABTS)	TAS ↑ (in plasma)	
				TBARS method: α-Tocopherol and GSH in tissue homogenates were measured by an HPLC-based method	MDA ↓ (in heart, liver, and brain)	
				α-Tocopherol and GSH in tissue analyzed with HPLC	GSH level ↑ (in heart and brain)	
					α-tocopherol ↑ (in heart, liver, and brain)	
				Antioxidant enzyme activities in organs	Catalase ↑ and GPx ↑ activities (in heart, liver, and brain)	
		4 g of the water extract of *G. paraguayense*	Participants with metabolic syndrome (*n* = 54)	Blood pressure and fasting blood glucose levels	SBP ↓ and, fasting blood glucose ↓	[29]
				Lipid profile	LDL-C ↓, TC ↓ (*p* = 0.08), TG ↓, HDL-C ↑	
				Activities of SOD and CAT	Activities of SOD ↑ and CAT ↑	
5	Antioxidant activity	Extract of leaves in 50% ethanol	Male Wistar rats; oxidative stress induced by t-BHP	TBA-reactive substance (TBARS) method	MDA level ↓ (in heart tissues)	[33]
				SOD activity was determined spectrophotometrically	SOD activity ↑ (in liver tissue)	
		Extract of leaves in 50% ethanol, 0.25 g/100 g BW for six weeks	CCl_4_-induced oxidative stress in Sprague–Dawley (SD) rats	HPLC-based method was used to measure levels of vitamin C, vitamin E, and GSH	Vitamin C ↑, vitamin E ↑, and GSH ↑	[15]
				Serum TAS by kit-based method	TAS ↑	
				MDA and GSH levels and GPx, SOD, CAT, and GST activities in liver	GPx ↑, SOD ↑, and CAT ↑, and GST ↑	
		100 g of *G. paraguayense* was provided for eight weeks	18 subjects suffering from hypercholesterolemia	Plasma levels of MDA, ascorbic acid and α-tocopherol	Plasma MDA ↓, ascorbic acid ↑, α-Tocopherol ↑	[28]
				Activities of GSH, GPx, SOD, and CAT in erythrocytes	GSH ↑, GPx ↑, and CAT ↑	
		Water extract of leaves, 4 g/day for 12 weeks	MS subjects (26 treatment, 28 placebo)	Red blood cell (RBC) SOD and CAT activity	SOD ↑ and CAT ↑	[8]
				Particle-enhanced immunonephelometry with an image analyzer and ELISA	CRP ↓, IL-6 ↓, TNF-*α* ↓	
6	Hepatoprotective activity	Water extract of *G. paraguayense,* 50 to 300 mg/kg BW	SD rats; CCl_4_-induced hepatotoxicity	Serum biochemical assays; BUN, CRE, ALT, and AST of rat serum were determined	TC ↓, TG ↓, ALT ↓, and AST ↓	[10]
				GSH levels in liver	GSH level ↑	
				Antioxidant enzyme activity	GPx ↑, SOD ↑, CAT ↑, and GR ↑	
				MDA levels in liver	MDA levels ↓	
				Immunoassay for TNF-α expression	TNF- α ↓	
				Histopathology	Damage to liver tissues ↓	
		80% ethanolic extract of leaves (1.4 g/kg) for 6 weeks study	Dimethylnitrosamine (DMN)-induced liver fibrosis in SD rats	Histopathology	BW ↑, LW ↑, necrosis ↓ and inflammatory effects ↓	[37]
		Various doses of *G. paraguayense* for 5 days (from days 5 to 10)	Immunocytochemical (IHC) staining analysis of the activation of cultured rat HSCs.	IHC staining of α-SMA expression and stress fiber formation in liver	α-SMA expression ↓ and stress fiber formation ↓	
			Culture of HSCs	Culture of HSCs	α-SMA expression ↓ and collagen 1 ↓	
				RT-qPCR and microarray for expressions of known and novel genes of liver damage	*Tgfb1* ↓, *Timp1* ↓, *Pparg* ↑, *Btg2* ↓, *Egr1* ↓, *Oldr1* ↓, *Nrg1* ↓, and *Hmgcs1* ↑	
		80% ethanolic extract of leaves, 10–100 μg	HSCs isolated from the liver of SD rats	Survival of HSCs	Survival of HSCs ↓	
				Microarray analysis	64% of 254 liver damage-related genes were restored	
		Methanolic extract of leaves, 400 mg/kg per day for six weeks	DMN-induced liver fibrosis in SD rats	Weight of body and organs and survival rate were calculated	BW ↑, LW ↑, SW ↓ and survival rate ↑; Necrosis ↑ and inflammatory effects ↑	[39]
		Methanolic extract of leaves, 400 mg/mL	Cultured HSCs of SD rats	Caspase activity assay	Activity of caspase-2, 3, 8, and 9 ↑	
				WB	Bcl-2 ↓, Mcl-1 ↓, Bax ↑, and Fas ↑	
		250, 500, 750, and 1000 μg/mL of 30DE	HSC-T6	MTT assay	IC_50_: 366 μg/mL	[40]
		5, 25, 50, 75, and 100 μg/mL of 30DE3	HSC-T6 and LX-2 cells	MTT assay	IC_50_: 20.8 and 22.5 μg/mL for HSC-T6 and LX-2 cells, respectively (30DE3)	
		5, 10, and 15 μg/mL of 30DE3	HSC-T6 cells	Wound healing assay and Transwell invasion assays for studying migration/invasion	Migration/invasion of HSC-T6 cells ↓	
		Water extract of *G. paraguayense*	Male Sprague–Dawley rats	Serum biochemical assays	ALT ↓ and ALP ↓	[41]
				Immunoassay of serum samples	IL-6 ↓ and TNF-α ↓	
				WB	TGF-1 ↓	
				RP-HPLC coupled with UV detection in serum and liver	MGL ↓	
			Male 5-week-old C57BL/6J mice	Length of large intestine	Length of large intestine ↑	
				WB	Occluding ↑, claudin-1 ↑	
				Intestinal microflora by PCR analysis	Bacteroidetes/Firmicutes ↑	
7	Anti-diabetic activity	Ethanolic extract of leaves, 300 mg/kg BW, administered by intraperitoneal injection for 12-weeks	C57BL/6 mice	Oral glucose tolerance test (OGTT)	Glucose levels ↓	[46]
				Glucose uptake of hepatic cells	Glucose uptake of hepatic cells ↑	
				Immunohistochemistry (IHC) staining	Number/area of islet cells and insulin levels ↑	
				WB	C/EBPβ ↓, PPAR-γ ↑, and PDX-1 ↑	
				Assay for lipid peroxidation products	MDA ↓,	
				Assay for glutathione (GSH)	GSH ↑	
8	Anti-airway-inflammation activity	Ethanol extract of leaves, 50 and 200 mg/kg	Ovalbumin-sensitized BALB/C Mice	Histopathology of lungs	Infiltration of inflammatory cells into lung tissues ↓	[57]
				TLC and DLC of bronchoalveolar lavage fluid (BALF)	Total cells ↓, eosinophils ↓, lymphocytes ↓, and neutrophils ↓	
				BALF	IL-4 ↓, IL-5 ↓, and IL-13 ↓	
				Flow cytometry	T cells (CD4 and CD8) ↓	
				PAS staining of lung sections	Mucus production ↓	
				Immunohistochemistry of lung tissues	NRF2 ↑	
				RT-qPCR	ICAM-1 ↓, VCAM-1 ↓, GCL ↑, and GST ↑	
				Thiobarbituric acid-reactive substance (TBARS) assay	GSH ↑ and generation of TBARS↓	
				Serum levels of IgE	IgE ↓	
				WB	GCL ↑ and GST ↑ GATA3 ↓ (in CD4 T cells and blood monocytes)	

BW: body weight; LW: liver weight; SHRs: spontaneously hypertensive rats; AST: aspartate aminotransferase; ALT: alanine aminotransferase; LDH: lactate dehydrogenase; BUN: blood urea nitrogen; CRE: creatinine; GSH: reduced glutathione; PCR: polymerase chain reaction; RP-HPLC: reverse-phase high-performance liquid chromatography; UV: ultraviolet; WB: Western blot; ↑: upregulation; ↓: downregulation/inhibition.

## 7. Discussion

The multiple pharmacological activities of *G. paraguayense* have been established in different in vitro, cell line, animal, and clinical studies. However, the different pharmacological activities of *G. paraguayense* have specific challenges in developing *G. paraguayense* as a supplement and/or therapeutic. Phytochemicals are a major factor for the different pharmacological activities; thus, their optimal content in *G. paraguayense* may enhance pharmacological activities. Environmental and genetic factors are key factors that can influence phytochemical content in plants [58,59,60]. However, the role of environmental conditions for morphological changes in *G. paraguayense* has been studied with other Mexican succulent plants [61]. Still, limited studies have been conducted to analyze the impact of environmental conditions on phytochemical content in *G. paraguayense.* Changes in environmental conditions may also explain the basis of variation in the pharmacological activities found in different studies. Similarly, the standardization of phytochemical analysis that will provide optimal phytochemical content and subsequently optimal bioactivities is suggested for future studies.

The studies have shown the correlation of phytochemical content with the pharmacological activities of *G. paraguayense,* which suggests that high phytochemical content can maximize phytochemical activities. Thus, genetic improvement in plants for high phytochemical content may pursued by plant breeders to produce optimized plants for pharmacological activities.

The anticancer activities of plants are the preferred activities for development as natural products are believed to be less toxic as compared to synthetic drugs. Additionally, the anticancer activities of *G. paraguayense* were found to be effective against different cancers such as the liver, colon, and skin, which again emphasizes its need to be developed with priority [6,20,21]. In anticancer activities, *G. paraguayense* was found to target crucial targets that are important in different cancers. However, studies on skin cancer and colon cancer activities are in early phases, i.e., studied only through corresponding cell line analyses [6,21]. It is suggested that the anticancer activities against skin and colon cancer must be validated through in vivo studies before pursuing clinical studies.

The anti-AD activities of *G. paraguayense* were apparent in different cell line studies, which revealed the inhibition of both Aβ (Aβ40 and Aβ42) as well as Tau phosphorylation, the major factors important in AD pathology [24]. The animal study also showed effective anti-AD activity through the activation of the AMPK signaling pathway [9]. Still, limited studies on extract components responsible for anti-AD activity have been conducted [9]. Precise information about the component of *G. paraguayense* extract responsible for anti-AD activity is required for optimal activity and the development of *G. paraguayense* as an anti-AD candidate.

The antihypertensive activity of *G. paraguayense* is associated with the inhibition of key antihypertensive target ACE [7]. Antihypertensive activity was observed in in vitro, in vivo, as well as clinical studies [27,29]. The optimization of antihypertensive activity may be the main factor that must be addressed before further developing *G. paraguayense* as an antihypertensive candidate. Further studies for identifying the components of *G. paraguayense* extract responsible for antihypertensive activity are strongly suggested to achieve optimal antihypertensive activity before further clinical studies.

The antioxidant activity of *G. paraguayense* was strongly evident in various types of in vitro methods based on different properties such as reducing power, different types of radical scavenging (DPPH, ABTS, and superoxide), and the inhibition of lipid peroxidation [5,19]. The antioxidant activity of *G. paraguayense* was also validated through cell lines and animal studies [12]. In different studies, the antioxidant activity of *G. paraguayense* was found to be correlated with TPC, TFC, and TAC, which gives an idea about the phytochemicals responsible for antioxidant activities [12,19]. Still, precise information on the antioxidant compound of *G. paraguayense* needs to be studied, which would be helpful for using *G. paraguayense* as a supplement and/or therapeutic against important diseases.

The anti-inflammatory activities of *G. paraguayense* have been observed in different cell lines and animal models induced with different inflammatory agents such as LPS and HFD [39,41]. Multiple studies have suggested that *G. paraguayense* suppresses the expression of inflammatory cytokines, including TNF-α and IL-6 [10,39,41].

Like antioxidant properties, the anti-inflammatory activity of *G. paraguayense* is believed to support other pharmacological activities of *G. paraguayense* [10,39,41]. It is also the main reason for the protective effect of *G. paraguayense* in different organs, including the colon, liver, brain, pancreas, and lungs, as observed in different animal studies. The anti-inflammatory activity of *G. paraguayense* can be protective toward other important organs such as the kidneys, heart, muscles, and bones. Thus, it is strongly suggested that the protective effect of *G. paraguayense* on these organs be studied in the future.

The tyrosinase inhibitory activity of *G. paraguayense* is the property that makes it important in different industries including food, agriculture, medicine, and cosmetics [49]. However, the tyrosinase inhibitory activity of *G. paraguayense* has not been studied through in vivo experiments, yet this is required in order to develop *G. paraguayense* as a candidate for skin whitening or other melanin-associated disorders. Further research is required to study the effect of *G. paraguayense* in vitro and in vivo melanin formation.

Effective activity of *G. paraguayense* was also observed against infectious diseases, including antiviral and antibacterial activities. The use of its antiviral activity against HBV was inspired by the traditional usage of *G. paraguayense* against liver-associated disorders. However, antiviral activities against HSV were also observed through the inhibition of HSV reproduction in Vero cells. The antibacterial activity against both Gram-positive and -negative bacteria was effective against Gram-positive bacterial strains. Effective anti-biofilm activity of the extract was also observed against MRSA, which supports the antibacterial candidature of *G. paraguayense*. Further, studies deciphering the mechanism of action behind the antiviral and antibacterial activities of *G. paraguayense* are suggested to develop it as an antimicrobial agent. Multiomics studies including genomics, transcriptomics, proteomics, and metabolomics have the potential to reveal comprehensive mechanisms behind the molecular actions of *G. paraguayense* that are suggested to achieve the optimal potential pharmacological activities of *G. paraguayense*.

In molecular studies, *G. paraguayense* was found to act on important signaling pathways such as the MAPK, AMPK, SMAD-dependent, and PI3K/AKT-dependent signaling pathways, which supports its strong and wide pharmacological activities.

In terms of liver-protective activity, *G. paraguayense* suppressed the MAPK and SMAD-dependent signaling pathways by inhibiting the key molecules of these pathways, including p-P38/P38, p-MEK, p-SMAD2/SMAD2, and p-SMAD3/SMAD3. However, MAPK- and SMAD-dependent signaling pathways are also targeted in other important diseases like cancer, diabetes, and obesity [62]. Thus, it is suggested that future studies be conducted on the anti-obesity effects and role of these pathways in the anti-diabetic and anticancer activities of *G. paraguayense*.

In the case of anti-AD activity, the important mechanism of *G. paraguayense* is the activation of the AMPK signaling pathway. The activation of the AMPK signaling pathway was observed through the enhancement in key components of the pathway, i.e., p-AMPK and AMPK in both in vitro cell lines and in vivo studies. AMPK pathway activation can be helpful against cancer and different metabolic syndromes, which again support the multiple pharmacological activities of *G. paraguayense* [63,64].

Similarly, the PI3K-/AKT-dependent signaling pathway was inhibited by *G. paraguayense* via anticancer activity through the suppression of p-AKT, which is the main regulator of the pathway in cancer. The level of PTEN was also found to be increased with the treatment, which is the negative regulator of PI3K-/AKT-dependent signaling. The PI3K/AKT pathway regulates different cellular processes, and studies have shown that activating the PI3K/AKT pathway can play a neuroprotective role [65].

Apart from antioxidant activity, in most of the studies, the phytochemicals/components of *G. paraguayense* important for pharmacological activities are not studied, thus posing a limitation on harnessing the optimum potential of the pharmacological activities of *G. paraguayense*. Thus, future studies analyzing the pharmacological activities of phytochemicals present in the extract of *G. paraguayense* are required.

Generally, clinical studies conducted to analyze the different activities of *G. paraguayense,* including antihypertensive, anti-inflammatory, and antioxidant activities, have been preliminary. These studies were conducted on small population sizes, which restricts the further development of *G. paraguayense* as a supplement/drug candidate on a large scale. In multiple studies, future clinical studies on large sample sizes with optimized doses have been proposed.

*G. paraguayense* with sorafenib showed strong synergy in their anticancer activity, which was higher than that of individual treatment. Synergistic approaches for other pharmacological activities of *G. paraguayense* have not been studied, and this can be considered as a gap. Therefore, synergistic approaches for studying other important pharmacological activities with conventional treatment is suggested for future studies.

Safety studies have also supported the therapeutic potential of *G. paraguayense* in different toxicity studies. However, studies on side effects and toxicity for the long-term use of *G. paraguayense* are still needed as different activities against such as liver-associated disorders, hypertension, and diabetes are chronic diseases that may require long-term medication. Additionally, the bioactive phytochemicals, including genistein, gallic acid, oxalic acid, and quercetin, reported in *G. paraguayense* are considered safe. Still, in high doses or/and long-term usage, they may cause adverse effects on the reproductive system [66] and cause anemia [67], kidney stone formation [68], and nephrotoxicity [69].

Therefore, studies analyzing the long-term safety of *G. paraguayense* usage in chronic diseases can conducted in the near future. *G. paraguayense* is largely considered a plant with multiple pharmacological properties, supported with strong antioxidant and anti-inflammatory activities. However, its development is in different stages depending on property. *G. paraguayense* can be further studied through proposed in vitro, in vivo, and clinical experiments for its development against important diseases.

## 8. Conclusions

The different pharmacological activities of *G. paraguayense* are in various phases of development, which may require different types of research efforts to develop it as a therapeutic and/or supplement. Climatic factors and genetic improvement are common gaps that may be considered for superior phytochemical content, which can help optimize the different pharmacological activities of *G. paraguayense*. *G. paraguayense* acts on crucial pathways that are associated with other diseases, suggesting that studies may be extended to these other linked diseases, including obesity.

## Data Availability

Data are contained within this article.
